# Influence of Model Grid Size on the Estimation of Surface Fluxes Using the Two Source Energy Balance Model and sUAS Imagery in Vineyards

**DOI:** 10.3390/rs12030342

**Published:** 2020

**Authors:** Ayman Nassar, Alfonso Torres-Rua, William Kustas, Hector Nieto, Mac McKee, Lawrence Hipps, David Stevens, Joseph Alfieri, John Prueger, Maria Mar Alsina, Lynn McKee, Calvin Coopmans, Luis Sanchez, Nick Dokoozlian

**Affiliations:** 1 Department of Civil and Environmental Engineering, Utah State University, Logan, UT 84322, USA;; 2 U. S. Department of Agriculture, Agricultural Research Service, Hydrology and Remote Sensing Laboratory, Beltsville, MD 20705, USA;; 3 Complutum Tecnologías de la Información Geográfica (COMPLUTIG), 28801 Madrid, Spain;; 4 Plants, Soils and Climate Department, Logan, UT 84322, USA;; 5 U. S. Department of Agriculture, Agricultural Research Service, National Laboratory for Agriculture and the Environment, Ames, IA 50011, USA;; 6 E & J Gallo Winery Viticulture Research, Modesto, CA 95354, USA;; 7 Department of Electrical Engineering, Utah State University, Logan, UT 84322, USA;

**Keywords:** evapotranspiration (*ET*), *GRAPEX*, *sUAS*, remote sensing, Two Source Energy Balance model (*TSEB*), contextual spatial domain/resolution, data aggregation, eddy covariance (*EC*)

## Abstract

Evapotranspiration (*ET*) is a key variable for hydrology and irrigation water management, with significant importance in drought-stricken regions of the western US. This is particularly true for California, which grows much of the high-value perennial crops in the US. The advent of small Unmanned Aerial System (*sUAS*) with sensor technology similar to satellite platforms allows for the estimation of high-resolution *ET* at plant spacing scale for individual fields. However, while multiple efforts have been made to estimate *ET* from *sUAS* products, the sensitivity of *ET* models to different model grid size/resolution in complex canopies, such as vineyards, is still unknown. The variability of row spacing, canopy structure, and distance between fields makes this information necessary because additional complexity processing individual fields. Therefore, processing the entire image at a fixed resolution that is potentially larger than the plant-row separation is more efficient. From a computational perspective, there would be an advantage to running models at much coarser resolutions than the very fine native pixel size from *sUAS* imagery for operational applications. In this study, the Two-Source Energy Balance with a dual temperature (*TSEB2T*) model, which uses remotely sensed soil/substrate and canopy temperature from *sUAS* imagery, was used to estimate *ET* and identify the impact of spatial domain scale under different vine phenological conditions. The analysis relies upon high-resolution imagery collected during multiple years and times by the Utah State University *AggieAir™ sUAS* program over a commercial vineyard located near Lodi, California. This project is part of the USDA-Agricultural Research Service Grape Remote Sensing Atmospheric Profile and Evapotranspiration eXperiment (*GRAPEX*). Original spectral and thermal imagery data from *sUAS* were at 10 cm and 60 cm per pixel, respectively, and multiple spatial domain scales (3.6, 7.2, 14.4, and 30 m) were evaluated and compared against eddy covariance (*EC*) measurements. Results indicated that the *TSEB2T* model is only slightly affected in the estimation of the net radiation (*R*_*n*_) and the soil heat flux (*G*) at different spatial resolutions, while the sensible and latent heat fluxes (*H* and *LE*, respectively) are significantly affected by coarse grid sizes. The results indicated overestimation of *H* and underestimation of *LE* values, particularly at Landsat scale (30 m). This refers to the non-linear relationship between the land surface temperature (*LST*) and the normalized difference vegetation index (*NDVI*) at coarse model resolution. Another predominant reason for *LE* reduction in *TSEB2T* was the decrease in the aerodynamic resistance (*R*_*a*_), which is a function of the friction velocity F_*_) that varies with mean canopy height and roughness length. While a small increase in grid size can be implemented, this increase should be limited to less than twice the smallest row spacing present in the *sUAS* imagery. The results also indicated that the mean *LE* at field scale is reduced by 10% to 20% at coarser resolutions, while the with-in field variability in *LE* values decreased significantly at the larger grid sizes and ranged between approximately 15% and 45%. This implies that, while the field-scale values of *LE* are fairly reliable at larger grid sizes, the with-in field variability limits its use for precision agriculture applications.

## 1. Introduction

Evapotranspiration (*ET*) is a key factor in the hydrologic cycle and in irrigation demand. Conventional methods for estimating *ET*, such as lysimeters and flux towers, are limited to sampling small areas on the order of 10^1^ to 10^3^ m^2^. For that, a more efficient method is needed as *ET* varies spatially under different micro-meteorological and vegetative conditions. Accordingly, spatially distributed data are important for mapping *ET* variations over large areas, particularly in agricultural regions containing many of crop types and growth stages. In recent decades, remote sensing products from various platforms and at various spatial resolutions have been applied in modeling different environmental processes (e.g., surface energy fluxes, water and carbon balance, net primary productivity) [1]. Improved sensor systems and methods in remote sensing, and particularly the advent of small unmanned aerial systems (*sUAS*), have made these technologies a valuable source of spatial information for *ET* estimation at the canopy level. *sUAS* can offer spatial coverage with sub-meter-resolution imagery for mapping canopy and soil temperature, which are the key surface states for estimating *ET* [2]. While satellites are characterized by either coarse resolution and high temporal frequency or by high spatial resolution and low repeatability [3], *sUAS* technology, in addition to offering high-resolution data [4–6], can be described as “flexible on timing” [7]. This means that remotely sensed information can be obtained when needed or on demand using *sUAS*. For these reasons, various methods are under development to employ *sUAS* data for *ET* estimation [2].

Remote sensing is a valuable source for accessing land surface spatial information [8]. Nonetheless, spatial scaling is recognized as a challenging issue, particularly in surface-atmosphere exchange [8,9], environmental modeling, and agricultural management [10] applications and research. Previous studies by Brunsell and Gillies [11] and Giorgi [12] indicated that spatial scaling becomes more complex in cases of heterogeneous land surfaces, and homogeneity is less likely to be met in reality [13]. Various models have been developed to describe aerodynamic or energy balance fluxes, but these models assume homogeneity in terms of agricultural type, surface roughness, surface temperature, and meteorological condition [13,14]. Heat fluxes, including latent heat flux (*LE*) and sensible heat flux (*H*), are highly influenced by land surface heterogeneity [15]. Therefore, the variability in land cover within a pixel or model grid size can result in significant error in the mean pixel or grid heat flux estimation [16]. Vegetated areas with partial canopy cover will have underlying soil/substrate affecting the remotely sensed data, and hence, require models that explicitly consider the different effects of these two sources on energy exchange and sensor integration [2]. Typically, remotely sensed data at different resolutions are employed as an approximation to describe the spatial variability of the interaction between surface and atmosphere [11]. Current and future developments in remote sensing, with information spanning from sub-meters to kilometers, are making upscaling (data aggregation) a crucial issue in scientific and methodological advances. This is particularly true for understanding the physics behind climate, weather, and the surface energy balance [13,17].

In general, spatial aggregation can be performed under two different procedures: forcing inputs to a coarser resolution or aggregating the derived fluxes from initial high-resolution data (contextual spatial domain). Long et al. [18] pointed out that forcing spatial data aggregation from Landsat bands to MODIS (Moderate Resolution Imaging Spectroradiometer) resolution results in different statistical and spatial properties in *ET* estimates than at the original Landsat resolution. Study cases of *LE* resulted in inaccuracies [19,20] due to a reduction in surface variability at MODIS scale [11]. Moreover, the structure of vegetation and aerodynamic roughness influence the aggregation of turbulent fluxes and produce bias when MODIS data is used [15]. On the other hand, Bian and Butler [21] showed that low-resolution data could retain the statistical characteristics of the original data using specific aggregation techniques such as average and median. In addition, the spatial aggregation of *ET* inputs removes the effects of heterogeneity on the land surface. Still, scaling up energy fluxes from Landsat to MODIS scale is necessary in large-scale environmental models [22]. However, Landsat resolution is needed for validating modeled outputs using flux towers [23].

Several methods exist for spatial aggregation of *ET* data, but they are in the exploratory stage [24]. Ershadi et al. [14] demonstrated that *ET* results reduced by 15% when aggregating Landsat TM (Thematic Mapper) imagery by 50% using the Surface Energy Balance System (*SEBS*) model. The *ET* reduction was caused by the decrease in roughness parameterization [14]. This outcome was also supported by Brunsell and Gillies [11], who indicated that the land surface heterogeneity is highly influenced by the input forcing aggregation of Landsat TM data affecting the surface heat fluxes. In contrast, French et al. [25] found no significant difference in daily *ET* estimates when they used *METRIC* (Mapping EvapoTranspiration at high Resolution with Internalized Calibration) model and upscaled data acquired by aircraft to Landsat resolution. However, another study by Kustas and Norman [16] that used a detailed soil-vegetation atmosphere simulation model along with the thermal-based two-source energy balance model found that varying the degree of heterogeneity within a pixel, either in terms of surface roughness, moisture status, or a combination thereof, can have a significant impact on the pixel aggregated flux.

A key question related to data aggregation was raised by Su et al. [26]: “How does the level of aggregation affect surface energy fluxes as fluxes are aggregated from the resolution at which they are observed to the coarse grid cell size of the atmospheric model?”. The study conducted by Guzinskia and Nieto [27] aimed to estimate *ET* using a Two Source Energy Balance (*TSEB*) model. They reported that sharpening Sentinel 3 thermal imagery at 1-km pixel resolution to higher resolution (20 m) visible/near-infrared is indicative of the main issue of the lack of fine resolution thermal-IR (InfraRed) data for input to remote sensing-based *ET* models, particularly when applied to agricultural areas. In addition, Niu et al. [28] indicated that the *TSEB* model *ET* output using *sUAS* imagery gives more reliable estimates compared to coarse-resolution data because the model can separate between canopy and soil components. Moreover, most previous studies exploring the effects of sensor resolution on modeled *ET* have used semi-empirical models (e.g., Surface Energy Balance Algorithm for Land (*SEBAL*) model) [14], while physically-based *ET* models are required to quantify changes in the water and energy exchange due to changes in fractional vegetation cover, roughness, canopy structure, phenology, etc. that are occurring at plant scale [29]. In addition, it is common knowledge that vineyards and orchard fields do not have the same row spacing. The spacing varies from 6 ft to 12 ft for vineyards [30] and from 8 ft to 18 ft for orchards [31].

In the same context as the investigations discussed above on spatial resolution and surface heterogeneity, this study investigates the impact of grid-size resolution on *LE* outputs from *TSEB* model using the component soil/substrate and canopy temperature version (*TSEB2T*) model applied to a complex agricultural canopy, namely a vineyard in California’s Central Valley. The study directly quantifies the effect of sensor resolution on key *TSEB* model inputs (i.e., land surface temperature (*LST*), leaf area index (*LAI*), canopy height (*h*_*c*_), canopy width-to-height ratio (*w*_*c*_/*h*_*c*_), and fractional cover (*f*_*c*_)) for estimating surface energy balance/*ET*. High-resolution optical and thermal data were acquired by an *sUAS* platform for vine and cover crop phenological stages at several different times during the day. In this research effort, the topics investigated include determining (a) whether the separation between canopy and soil/substrate temperature (*T*_*c*_ and *T*_*s*_, respectively) using *TSEB2T* is valid for coarse spatial domains (e.g., towards Landsat scale); (b) the effect of spatial resolution of *TSEB2T* inputs on the magnitude and spatial variation of *LE*; (c) if the different spatial domain scales/pixel resolutions under study (3.6, 7.2, 14.4 and 30 m) have an impact on the magnitude of the *LE* and quantify the discrepancies as a function of resolution.

### 1.1. TSEB2T Model

*TSEB2T* is a physically based approach developed by Norman et al. [32] that explicitly accommodates the difference between aerodynamic and radiometric surface temperature that affect the radiative and convective exchange of energy between soil and canopy systems and the lower atmosphere. The main concept underpinning the *TSEB2T* approach is modeling of the partitioning of radiative and turbulent energy fluxes between canopy and soil systems. In this case, *H* is partitioned between soil and canopy, which is dependent mainly on *T*_*c*_ and *T*_*s*_ differences with the overlying atmosphere and their respective aerodynamic coupling.

**Figure 1.**
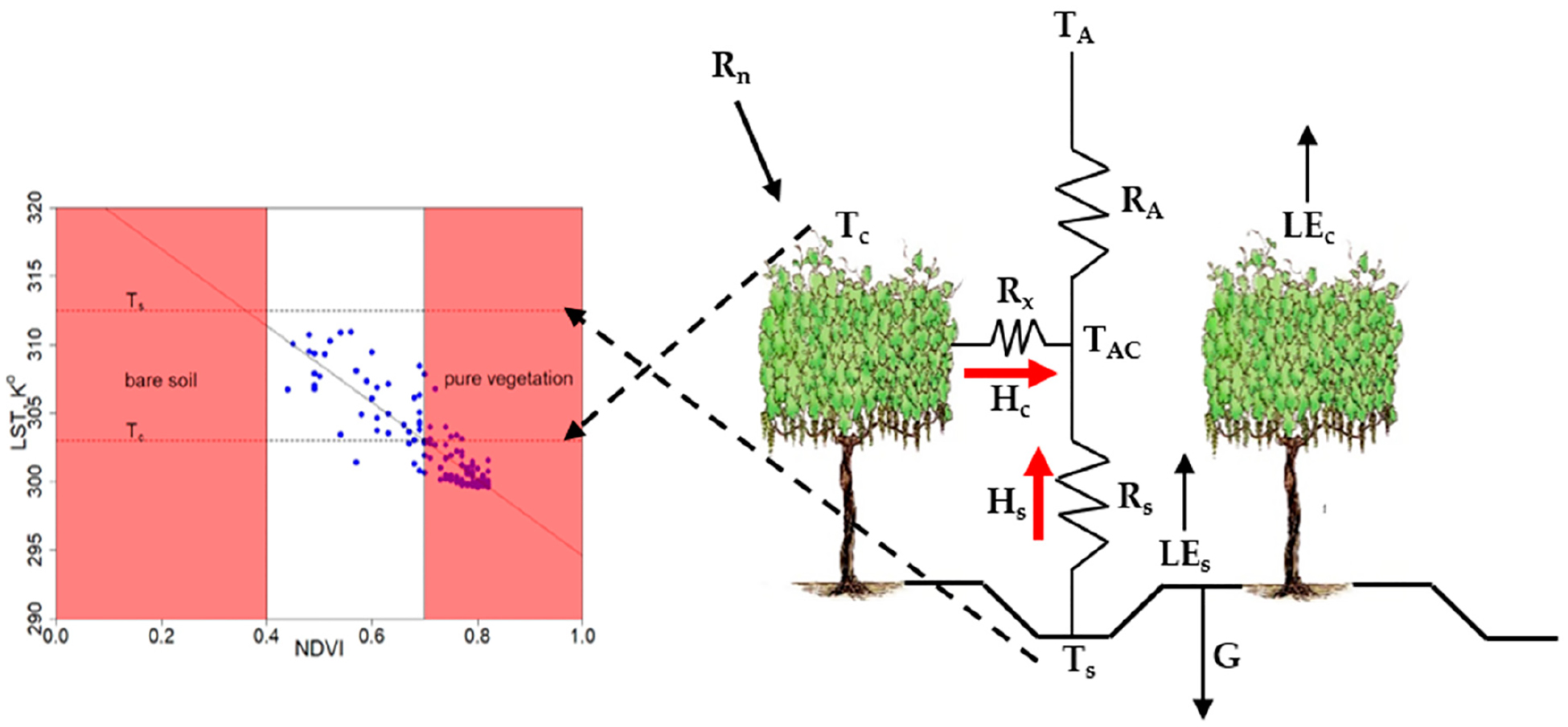
Schematic representation of *TSEB2T* model.

As shown in the Figure 1, the *TSEB2T* model separates the surface energy balance between soil and vegetation as follows:

(1) $$R_{n}=LE+H+G,$$

(2) $$R_{nc}=H_{c}+LE_{c},$$

(3) $$R_{ns}=H_{s}+LE_{s}+G,$$

where *R*_*n*_ is the net radiation, *H* is the sensible heat flux, *LE* is the latent heat flux, and *G* is the soil heat flux. All units of fluxes are in W/m^2^. Subscripts of *c* and *s* represent the canopy and soil components, respectively. Because *T*_*s*_ and *T*_*c*_ can be derived from the *LST* with a high enough resolution of optical data, energy fluxes (*R*_*n*_, *H*) can be calculated directly from the component temperatures (*T*_*c*_ and *T*_*s*_) and estimated aerodynamic resistances of canopy and soil components, while *G* is parametrized as a portion of soil net radiation (*R*_*ns*_). *LE*_*c*_ and *LE*_*s*_ are solved as residuals when (*T*_*c*_ and *T*_*s*_) observations are available.

(4) $$G=c_{G}R_{ns}$$

where *c*_*G*_ is an empirical coefficient changing over the daytime [2].

To estimate the sensible heat flux for vegetation and canopy, Norman et al. [32] proposed a series of soil-vegetation resistance network as illustrated in Figure 1:

(5) $$H=H_{c}+H_{s}=\rho_{air}C_{p}\frac{T_{AC}-T_{A}}{R_{A}}=\rho_{air}C_{p}\left[\frac{T_{C}-T_{AC}}{R_{x}}+\frac{T_{s}-T_{AC}}{R_{s}}\right]$$

(6) $$R_{A}=\frac{ln\left(\frac{z_{T}-d_{0}}{z_{0H}}\right)-\Psi_{h}\left(\frac{z_{T}-d_{0}}{L}\right)+\Psi_{h}\left(\frac{z_{0H}}{L}\right)}{\kappa^{\prime }u_{*}}$$

where ρ_*air*_ is the air density (kg/m^3^); *C*_*p*_ is the heat capacity of the air at constant pressure (J/(kg·K)); *T*_*c*_ and *T*_*s*_ are canopy and soil temperature (K), respectively; *T*_*AC*_ is the temperature of canopy-air space (K); and *T*_*A*_ is the temperature of air (K). *R*_*A*_ is the aerodynamic resistance to heat transport from the soil/canopy system (s/m), *R*_*x*_ is the boundary layer resistance of the canopy leaves (s/m), *R*_*s*_ is the aerodynamic resistance to heat transport in the boundary layer close to the soil surface (s/m), *z*_*T*_ is the measurement height for *T*_*A*_, *z*_0*H*_ is the roughness length for heat transport, *d*_0_ is the zero-plane displacement height (m), *L* is the Monin-Obukhov length (m), κ′ = 0.4 is the von Karman’s constant, u_*_ is the friction velocity (m/s), and Ψ_*h*_ is the adiabatic correction factor for the momentum.

Key factors, including *T*_*s*_ and *T*_*c*_, *LAI*, f_c_, *w*_*c*_/*h*_*c*_, and *h*_*c*_, are required as inputs for the *TSEB* model to parameterize the radiative and convective flux exchanges between soil/substrate and canopy. Other parameters related to micro-meteorological data are also needed to run the model. In the study conducted by Chirouze et al. [33] comparing different remote sensing *ET* models, results indicated that *TSEB* is a better model for *ET* estimation compared to others, being less sensitive to roughness parameters. This lack of sensitivity to roughness parameters was also recently verified for vineyards by Alfieri et al. [34]. The *TSEB* model has been extensively tested for years over agroecosystems [35–37], natural ecosystems [38,39], and wetlands [40,41].

*TSEB2T* was originally developed and evaluated by Kustas and Norman [42] using multiple thermal-IR radiometer viewing angles and was further refined and tested by Nieto et al. [2] applied to high resolution imagery from *sUAS* or other airborne sources. They found that *TSEB2T* gave better agreement with tower fluxes compared to other versions of *TSEB*, including *TSEB-PT* (Priestly-Taylor), *TSEB-DTD* (Dual-time-difference), and *TSEB-2T-DMS* (Data-mining sharpening of temperature). *TSEB-PT* is one version of the *TSEB* model that assumes a composite radiometric temperature (*T*_*rad*_) containing temperature contribution from the canopy and soil/substrate, which is typically provided by the radiometer. The decomposition of radiometric temperature (*T*_*rad*_) between plant canopy and soil/substrate is based on *f*_*c*_. *TSEB-DTD* is a further development of the *TSEB-PT* model described by Norman et al. [43]. The *TSEB-DTD* model is similar to the *TSEB-PT* model in that it divides the composite *T*_*rad*_ into *T*_*c*_ and *T*_*s*_. However, *TSEB-DTD* uses two observations of *T*_*rad*_: the first observation obtained 1.5 h after the sunrise (*T*_*rad*,0_) and the second one during the daytime (*T*_*rad*,1_). This version is less sensitive to errors in absolute radiometric surface temperature or the use of non-local air temperature observations. *TSEB-2T-DMS* partitions *T*_*s*_ and *T*_*c*_ using a data-mining fusion algorithm [44] to sharpen the original *LST* to be similar to the optical data, which would allow a better discrimination between *T*_*s*_ and *T*_*c*_.

The Nieto et al. [2] *TSEB2T* approach is a contextual *TSEB* that estimates *T*_*s*_ and *T*_*c*_ from composite *LST* imagery using the relationship between vegetation index (*VI*) and *LST* for extracting *T*_*s*_ and *T*_*c*_ within a spatial domain. *T*_*s*_ and *T*_*c*_ are calculated by averaging the temperature of pixels that are considered pure soil/substrate and pure canopy in a contextual spatial domain, namely, a two-dimensional plot of *LST* versus *VI*, such as Normalized Difference Vegetation Index (*NDVI*) (see Figure 1). That is, each pixel of the spatial domain is assigned based on *T*_*c*_ and *T*_*s*_ corresponding to the average temperature of the 0.6-m grids that are considered pure vegetation and bare soil, respectively. Both soil/substrate or canopy features are determined using *NDVI* threshold values (or any other vegetation index). The selection criterion for detecting the *NDVI* threshold of pure soil for bare soil interrows or, for most of the growing season, a soil senescent and cover crop stubble mixture (substrate) (*NDVI*_*s*_) can be further supported by other sources such as *NDVI* value from a *NDVI-LAI* curve when *LAI* in the interrows is nearly zero. The pure vine canopy *NDVI* threshold (*NDVI*_*v*_) can be calculated as the mean value of pixels identified as pure vegetation in a binary (soil-vegetation) classification of a multispectral image. In cases of very dense vegetation where pure soil pixels do not exist or sparse vegetation lacking pure vegetation pixels inside the spatial domain, a linear fit between *LST* and *NDVI* can be developed where *T*_*s*_ and *T*_*c*_ can be estimated by previously defining the *NDVI* thresholds of canopy and bare soil (Figure 1).

### 1.2. TSEB2T Main Inputs

#### 1.2.1. Leaf Area Index (*LAI*)

*LAI* is one of the key inputs in *TSEB* influencing the computation of *ET* as leaves distribution is the driving factor in energy and mass exchange in this model. *LAI* is also difficult to acquire using ground-based leaf-scale measurements, due to the time-intensive effort required [45], complications using indirect methods in complex canopies, and lack of any spatial extent for mapping, even at the field scale [46]. Therefore, considerable efforts have been devoted to developing remote sensing approaches to estimate *LAI* [47].

Estimating spatial distribution of *LAI* is challenging in vineyards, with their rows of vines and interrows with little to no vegetation. A previous study conducted by Johnson [48] evaluated the *LAI-NDVI* relationship in vineyards using IKONOS satellite imagery with 1-m pixel resolution and comparing *NDVI* to ground-based *LAI* measurements. They concluded that *LAI* can be computed from *NDVI* using simple linear regression for the vineyard they studied planted with red grape in six blocks of different planting density, trellis, age, and cultivar. In addition, Johnson et al. [48] and Dobrowski et al. [49] showed that remotely sensed indices of soil and vegetation can be used to estimate *LAI*. However, a study by Fang [50] indicated that limitations exit when using vegetation indices (*VIs*) to describe the spatially distributed *LAI* due to sensitivity of the *LAI-VIs* relationship to vegetation type and substrate/soil type, and hence, will not be stable or applicable over large areas. Indeed, operational satellite retrievals of *LAI*, particularly for vineyards [51], have a level of uncertainty that could affect modeling fluxes using *TSEB*. Furthermore, canopy phenological properties (i.e., chlorophyll content and average leaf angle), along with other factors such as atmospheric scattering, soil reflectance, and the effects of mixed pixel due to a composite of soil and vegetation that changes with time and from one place to another, affect the accuracy of *LAI* estimation [47]. To improve the *LAI-VIs* relationships, numerous studies have been conducted to estimate *LAI* using statistical approaches. Artificial Neural Network (*ANN*) was very promising and is simple to use [50]; however, this method does not allow for standardization of the *LAI* estimation [52]. As described by Gonsamo and Pellikka [53], there is currently no standard or arbitrary characteristic parameters, specific vegetation types, or data sources can be employed for *LAI* estimation. Thus, researchers must develop custom models by considering the sensitivity of parameters to *LAI* within an expected range [53].

#### 1.2.2. Canopy Height (*h*_*c*_)

**Figure 2.**
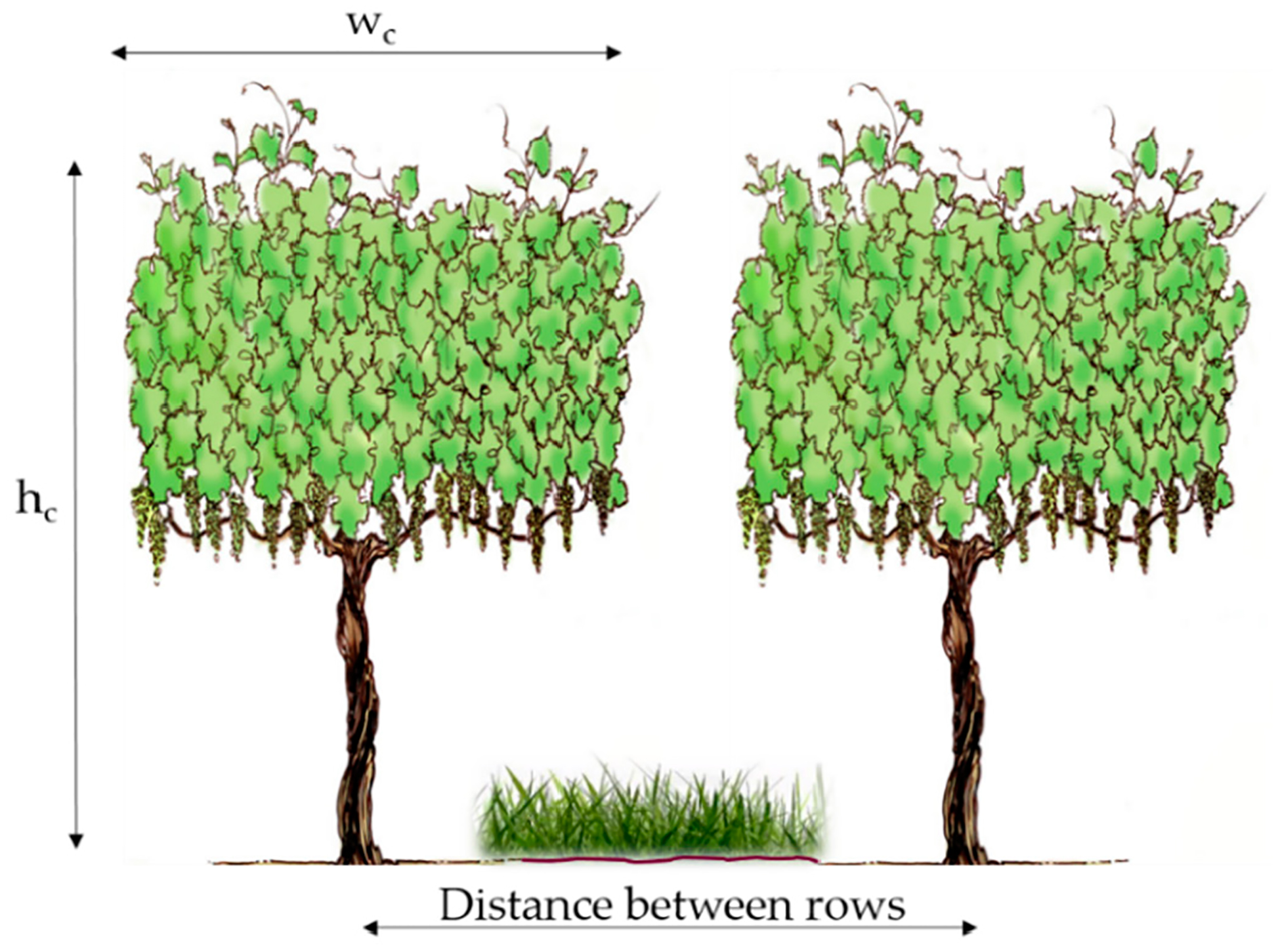
Schematic diagram for canopy *w*_*c*_/*h*_*c*_ ratio.

The *h*_*c*_ value is representative (mean) over the area of interest, but it can also be incorporated from spatial sources. An estimate of *h*_*c*_ can be produced using high-resolution images from *sUAS* and other airborne sources processed with structure-from-motion (*SfM*) methods in Agisoft or Pix4D, among others, along with digital elevation models (*DEM*) and point clouds (*LiDAR*). The value of *h*_*c*_ is required for the *TSEB2T* model to estimate surface aerodynamic roughness and radiation transmission in row crops and to calculate the foliage density, which are all required for the canopy wind attenuation model (Figure 2).

#### 1.2.3. Fractional Cover (*f*_*c*_) and Canopy Width (*w*_*c*_):

Fractional cover (*f*_*c*_) is the proportional area of vine for each spatial domain under analysis, where values vary from 0 through 1. *f*_*c*_ is used to estimate *w*_*c*_ and the clumping index, which is a factor to adjust the remotely sensed *LAI* value, which is assumed to be uniformly distributed (homogeneous) over the landscape instead of being clumped [54]. These are used to estimate the actual canopy gap fraction, which is greater than the homogenous case. It is required as input for the radiation transmission and wind extinction algorithms through the canopy layer. The magnitude of *w*_*c*_ is a length scale representing the area occupied by vine leaves along the vine row, which varies spatially and temporally based on phenology and management (i.e., vine manipulation via the trellis system and pruning) (Figure 2).

#### 1.2.4. *w*_*c*_/*h*_*c*_ Ratio

In *TSEB* and *TSEB2T* models, the *w*_*c*_/*h*_*c*_ ratio is required as input to the radiation transmission and wind extinction algorithms through the canopy layer developed for vineyards [2,55]. The *w*_*c*_/*h*_*c*_ ratio value is obtained by simply calculating canopy width over canopy height (Figure 2).

## 2. Materials and Methods

**Figure 3.**
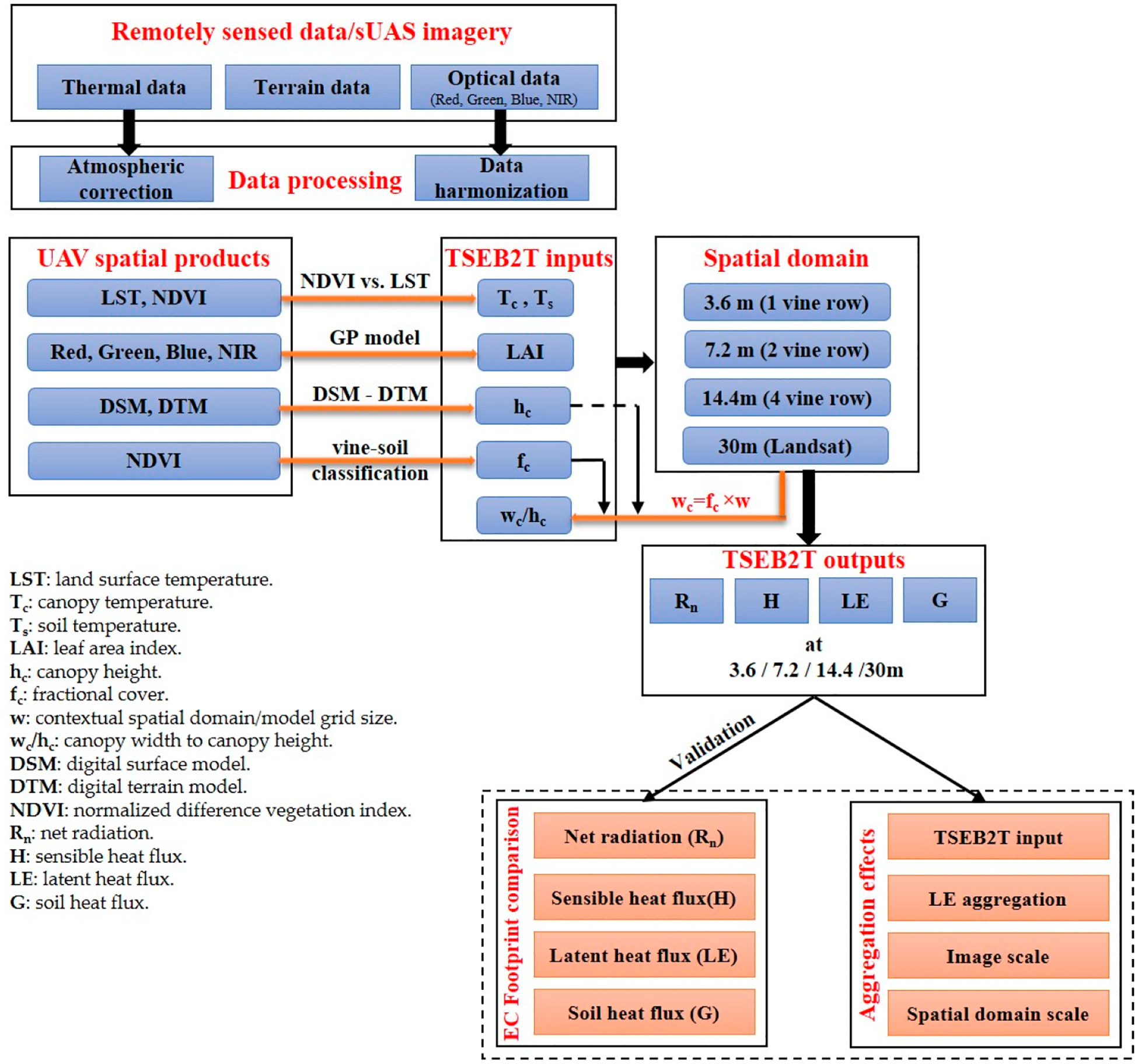
Study methodology for assessing the impact of the *TSEB2T* contextual spatial domain.

The methodology to assess the impact of changes in the contextual spatial domain for the *TSEB2T* model is graphically presented in Figure 3. The analysis was performed for wine grape growing seasons (May2013August) using different spatial domain scales.

### 2.1. Study Area and Data Sources

**Figure 4.**
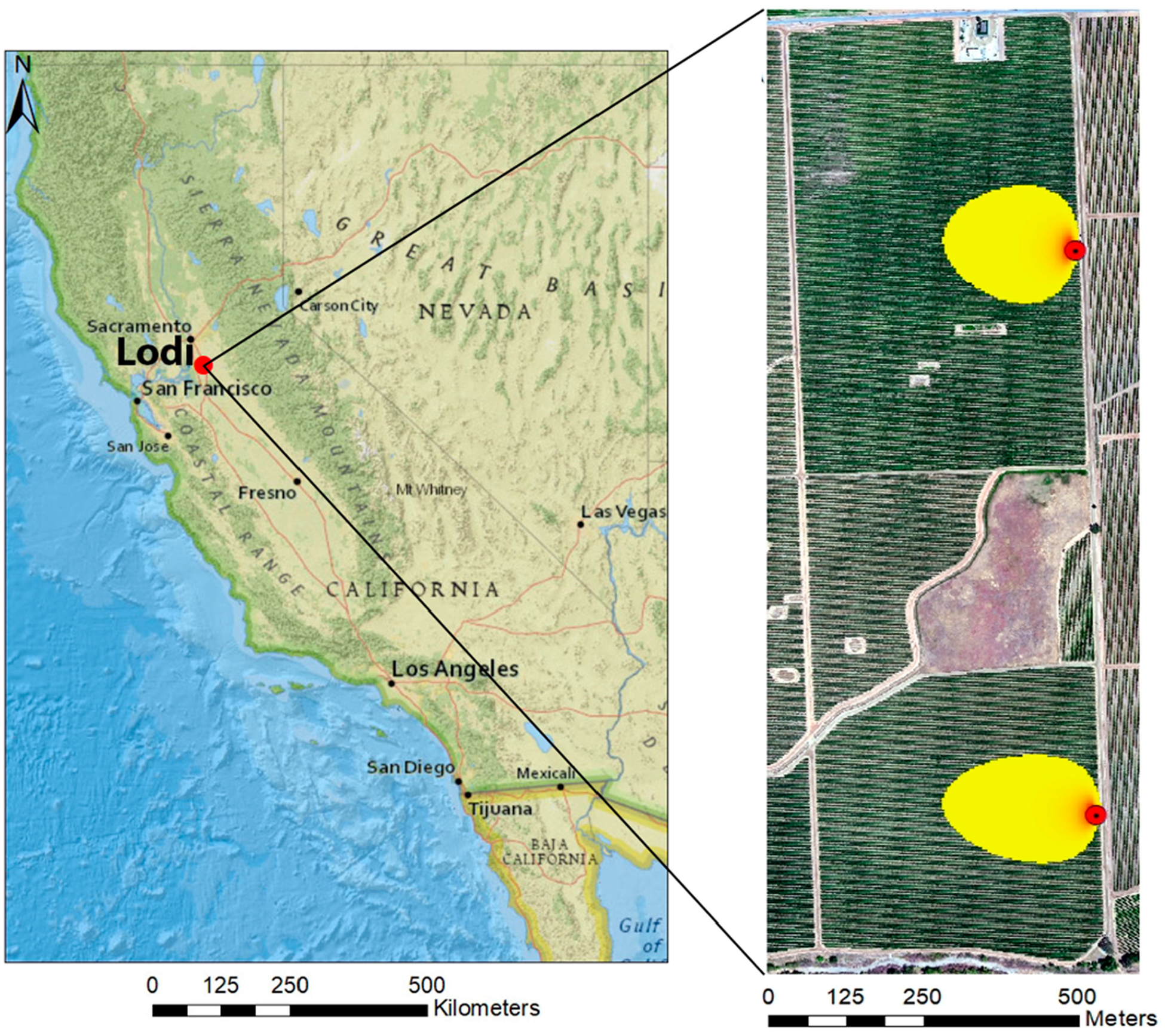
Layout of study area in Lodi, California, locations of *EC* towers and example of 90% of *EC* footprint at afternoon for 02 June 2015.

The study site is located near Lodi, California (38.29°N, 121.12°W) with an area of approximately 150 ha. The two vineyard blocks (north and south) are part of the Sierra Loma vineyard ranch (Figure 4). The north block was planted in 2009, while the south block was implemented in 2011, leading to different levels of vine maturity, and hence, biomass and grape production. Both vineyards are managed cooperatively by Pacific Agri-Lands Management. The plantation structure in both fields is the same, with vine rows having east–west orientation with a row width of 3.35 m (11 feet). A cover crop grows in the interrows, occupying ~2 m, with bare soil strips along the vine rows spanning ~0.7 m. The purpose of the cover crop is to deplete plant available water in the interrows from the fall and winter precipitation in order to control vine growth in the spring by irrigation. Typically, the vine height varies between 2 m and 2.5 m above ground level (*agl*) and vine biomass is concentrated mainly in the upper half of the vine canopy height. The actual vine canopy width varies spatially and temporally due to vine management practices. This study site is a part of the Grape Remote Sensing Atmospheric Profile and Evapotranspiration eXperiment (*GRAPEX*) project run by the USDA Agricultural Research Service in collaboration with E&J Gallo Winery, Utah State University, University of California in Davis, and others [56].

Flights campaigns were conducted by the *AggieAir sUAS* program at Utah State University (https://uwrl.usu.edu/aggieair/). Optical and thermal high-resolution imagery of the study site were collected from different flights in 2014, 2015, and 2016. Vegetative and soil conditions changed between the field campaigns. The 2016 flight imagery represents the early part of the growing season, around the time phenologically of fruit set, while other flights in 2014 and 2015 represent full vine canopy development and grape vine phenology in the pre- and post-veraison stages. Table 1 lists information concerning the different flights. The pixel resolution of the *sUAS* imagery collected is 10 cm and 60 cm for the optical and thermal bands, respectively. The spectral range of the optical data is similar to Landsat and includes visible bands (red, green, and blue) as well as near-infrared. However, the thermal band is different than Landsat, having a bandwidth spanning from 7 to 14 *μ*m [57]. Thermal data, acquired using a lightweight micro-bolometer camera, were radiometrically calibrated [58].

To evaluate the *ET* performance at different spatial domain scales, two eddy covariance (*EC*) flux systems were deployed for the measurements of turbulent fluxes, including *LE* and *H*, and the available energy terms of *R*_*n*_ and *G*. Both towers are located at the eastern edge of the fields, due to predominant winds from the west. Ground measurements, including soil temperature and soil moisture were also collected. A complete listing of all measurements on the towers is given by Kustas et al. [56]. Details of the post processing of the *EC* data as well as the available energy measurements are provided by Alfieri et al. and Agam et al. [59,60].

*EC* micrometeorological data also included wind speed, air temperature, vapor pressure, air pressure, and shortwave radiation. Hourly average values of these atmospheric forcing variables, as well as the components of the surface energy balance, were computed. Table 2 illustrates the in-situ micrometeorological parameters and the name of the instruments used for the measurements.

Given the high fluctuation of atmospheric conditions during the daytime, the flux footprint or contributing source area of each *EC* tower was estimated for the hourly period encompassing *sUAS* flight campaigns using the two-dimensional (*2D*) flux footprint model developed recently by Kljun et al. [61]. Because a 100% *EC* footprint fetch could extend over the study area, a 90% footprint area (90% cutoff) was used for analysis. Then, the weighted footprint area was divided by 0.9.

### 2.2. Data Processing

In this study, images were acquired remotely by *sUAS*, and the data were terrain corrected using georeferencing based on ground control points (*GCPs*). Furthermore, both thermal and optical data were atmospherically corrected.

#### 2.2.1. Thermal Data

Torres-Rua [57] indicated that the thermal data obtained from the *sUAS* thermal sensors in this study are adversely affected by changes in transmissivity and atmospheric radiance. For this reason, ground measurements of temperature were collected in the same timeframe as the *sUAS* flight and compared with the imagery to calibrate the thermal image data. More details about the calibration of temperature imagery related to this study can be found in Torres-Rua [57].

#### 2.2.2. Optical Data

Radiometric agreement between remotely sensed data from different platforms constitutes one of the major challenges in image processing. Therefore, in this research, the images acquired by *sUAS* were upscaled and harmonized with Landsat using the point spread function (*PSF*). More details related to *sUAS* data harmonization can be found in Hassan-Esfahani et al. [62].

### 2.3. Energy Balance Closure Adjustment Methods for EC

While the *EC* technique provides measurements of turbulent fluxes *H* and *LE*, a lack of energy balance closure with the available energy terms *R*_*n*_ and *G* [63] is well documented. This results in *R*_*n*_ − *G* > *LE* + *H* [64,65], and the computed closure ratio (*CR*) evaluates the energy balance discrepancy, *CR* = (*H* + *LE*)/(*R*_*n*_ − *G*). This ratio varies during the daytime, but for the *sUAS* flights [55] it was found to be above 0.8, except for the May 2 afternoon flight where it fell to around 0.7.

To avoid any bias when comparing the energy balance models with *EC* field measurements, the energy closure issue needs to be handled and resolved. Twine et al. [66] suggested a method for energy balance closure that assumes the Bowen ratio (*H*/*LE*) before and after adjustment are the same, while considering both *R*_*n*_ and *G* as reliable measurements. A modified *H* and *LE* can be calculated as:

(7) $$LE*=\frac{\left(R_{n}-G\right)}{\left(B+1\right)}$$

(8) $$H*=\frac{\left(R_{n}-G\right)}{\left(\frac{1}{B}+1\right)}$$

where LE* and H* denotes the closure adjusted latent and sensible heat flux, respectively.

### 2.4. Contextual Spatial Domain

The representative *TSEB2T* modeling grid size for the vineyard blocks was taken at 3.6 m, which corresponds to encompassing 6 × 6 grid or 36 *sUAS* thermal pixels having a resolution of 0.6 m. At this grid size, the inputs to *TSEB2T* incorporate the thermal-IR and optical bands of a vine row and adjusted interrows having a length scale of 3.35 m. Larger spatial domain scales were considered in this study, including 7.2 m, 14.4 m, and 30 m, to investigate the influence of domain size on the *TSEB2T* estimates. These selected values correspond to multiple vine rows spacing of 7.2 m (two rows), 14.4 m (four rows), and 30 m (Landsat scale—nine rows).

### 2.5. TSEB2T Inputs

The *TSEB2T* model developed by Nieto et al. [2] and implemented in Python language and is available at https://github.com/hectornieto/pyTSEB.

#### 2.5.1. Leaf Area Index (*LAI*)

To assess the spatial heterogeneity of *LAI*, an approach was developed in this study to calculate *LAI* using a genetic programming (*GP*) model using the Eureqa software. The *GP* model associated *sUAS* imagery and *LAI* ground measurements collected with an indirect method using (*LAI-2200*, *LI-COR*, Lincoln, Nebraska) plant canopy analyzer measurements at several locations within the northern and southern vineyards with additional validation using destructive *LAI* sampling at several locations [46]. Before performing the *GP* model calculations, imagery features were classified into two categories, vine and interrow, and then statistical calculations were separately carried out for the optical properties of each category. The main optical reflectance used in this analysis comprise the original bands (red (*R*), green (*G*), blue (*B*), and near-infrared (*NIR*)), along with two conventional *VIs* (*NDVI* and *NIR*/*R*). Statistical computations were performed using the fine-resolution data inside the spatial domain scales (3.6 m, 7.2 m, 14.4 m, and 30 m), which included the maximum, minimum, area, mean, standard deviation, and sum. The *GP* model integrates all of these corresponding statistics to construct a relationship to *LAI* observations.

#### 2.5.2. Canopy Height (*h*_*c*_)

Spatial data from the digital terrain model (*DTM*) [67] and digital surface model (*DSM*) were aggregated into multiple spatial scales by employing a simple averaging method; then, *h*_*c*_ was calculated using the expression: *h*_*c*_ = *DSM-DTM*. For example, in the case of a 7.2-m grid, the average values of *DSM* and *DTM*, *DSM*_(7.2)_, and *DTM*_(7.2)_, respectively, were computed inside the grid window, then the height of the canopy was computed as: $h_{c_{\left(7.2\right)}}=DSM_{\left(7.2\right)}-DTM_{\left(7.2\right)}$.

#### 2.5.3. Fractional Cover (*f*_*c*_) and Canopy Width (*w*_*c*_)

The north and south vineyard blocks were classified into two categories, vine and interrow, based on *NDVI*. Then the vine area inside each spatial domain was calculated and divided by the total area of the grid to calculate the *f*_*c*_. *w*_*c*_ inside each spatial domain was computed using *f*_*c*_ and the width of the grid (*w*) under analysis, i.e., *w*_*c*_ = *f*_*c*_ × *w*. To calculate the representative width of the vine canopy, the total width was rescaled and standardized at multiple spatial domain scales, depending on the number of rows inside each grid. For example, in the case of a 3.6-m grid, one vine row was counted inside, while in a 7.2-m grid, the number of rows was doubled.

#### 2.5.4. *w*_*c*_/*h*_*c*_ Ratio

*w*_*c*_/*h*_*c*_ was calculated by simply dividing canopy width by canopy height at each contextual spatial domain.

### 2.6. Goodness-of-Fit Statistics

Evaluating the performance of the *TSEB2T* model with the *sUAS* imagery for the four different modeling grid resolutions involved comparing the estimated fluxes with measurements from the *EC* towers. Computed statistical metrics included the root mean square error (*RMSE*), the normalized root mean square error (*NRMSE*), mean absolute error (*MAE*), mean absolute percentage error (*MAPE*), and Nash–Sutcliffe efficiency coefficient (*NSE*). A value of *NSE* = 1 indicates perfect agreement between modeled and observed flux, while *NSE* approaching 0 means that the agreement is very poor, and *NSE* < 0 indicates unacceptable performance [68]. These statistical measurements are calculated as follows using *LE* as the flux:

(9) $$\text{RMSE}=\sqrt{\frac{1}{N}\overset{N}{\underset{i=1}{\sum }}{\left(LE_{m,i}-LE_{o,i}\right)}^{2}}$$

(10) $$\text{NRMSE}=\frac{\text{RMSE}}{\sigma_{o}}$$

(11) $$MAE=\frac{\sum_{i=1}^{n}\left|LE_{m,i}-LE_{o,i}\right|}{n}$$

(12) $$\text{MAPE}=\frac{\sum_{i=1}^{N}\left|\frac{LE_{m,i}-LE_{o,i}}{LE_{o,i}}\right|*100}{n}$$

(13) $$NSE=1-\frac{\sum_{i=1}^{n}{\left(LE_{m,i}-LE_{o,i}\right)}^{2}}{\sum_{i=1}^{n}{\left(LE_{m,i}-{\bar{LE}}_{o,i}\right)}^{2}}$$

where *LE*_*m*_. denotes the modeled latent heat flux obtained from the *TSEB2T* aggregated up for the estimated flux footprint/source area, *LE*_*o*_ denotes the observed values from the *EC* tower, and *n* represents the number of observations, *σ*_*o*_ denotes the standard deviation of observed values.

*LE* was used for evaluating the impact of spatial resolution or grid size on modeled fluxes. At field scale, the evaluation is done using the spatial mean and coefficient of variation (*CV*) statistics. For *LE* statistical characteristics, frequency and cumulative distribution curves were used. Finally, to evaluate the effect of aggregating *LE* at 3.6 m, 7.2 m, 14.4 m, and Landsat scale, relative difference (relative error) was used. Relative difference (relative error) is defined as the root mean square error (*RMSE*) between the aggregated resolution and its reference grid size resolution of 3.6 × 3.6 m divided by the spatial mean (*μ*) value computed from the reference grid size (3.6 m × 3.6 m), i.e., *E*_*r*_ = *RMSE*/*μ* [14]. Each grid value of aggregated data was compared to the *n* × *n* set of reference scale or resolution (3.6 m) grid using *E*_*r*_.

## 3. Results and Discussion

### 3.1. TSEB2T Contextual Spatial Domains Validation

#### 3.1.1. *EC* Footprint Estimation

**Figure 5.**
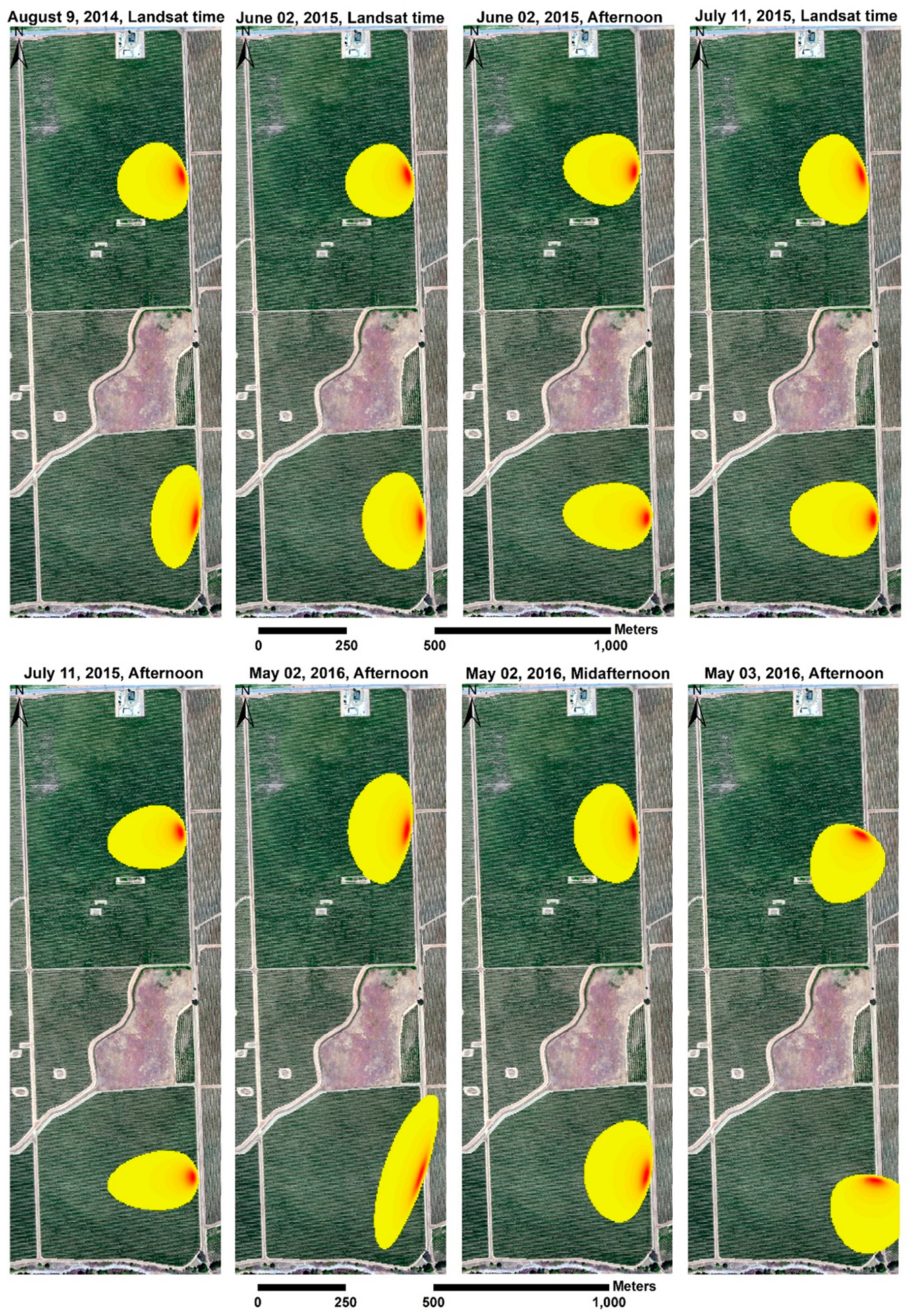
Layout of 90% *EC* footprints for two towers at different times considered by this study.

The results of footprint analysis using the *2D* flux model developed by Kljun et al. [61] and described in Section 2.1 are shown in Figure 5 for the different *sUAS* flights.

The orientation and size of each flux footprint/source area depends on the micro-meteorological conditions at the site measured by the *EC* towers, which include the turbulence fluxes, friction velocity (u_*_), and wind speed, which affect atmospheric stability, and canopy and *EC* measurement height, which affect the effective sampling height and wind direction that affects the orientation of the footprint. The total statistical weight of the footprint is taken to equal unity, although the actual area computed by the footprint model represents 90% of the contribution since the additional 10% essentially makes no measurable contribution. To compare the fluxes computed by the *TSEB2T* model at the different spatial resolutions with the *EC* measurements, the source area estimated by the footprint model was multiplied by the corresponding modeled fluxes (*R*_*n*_, *H*, *LE*, *G*) using *ArcGIS10.6*. Then, a comparison between the weighted fluxes at the different spatial resolutions or grid sizes from the *TSEB2T* version of *TSEB* and *EC* measurements was performed to assess model performance.

#### 3.1.2. Statistical Performance

**Figure 6.**
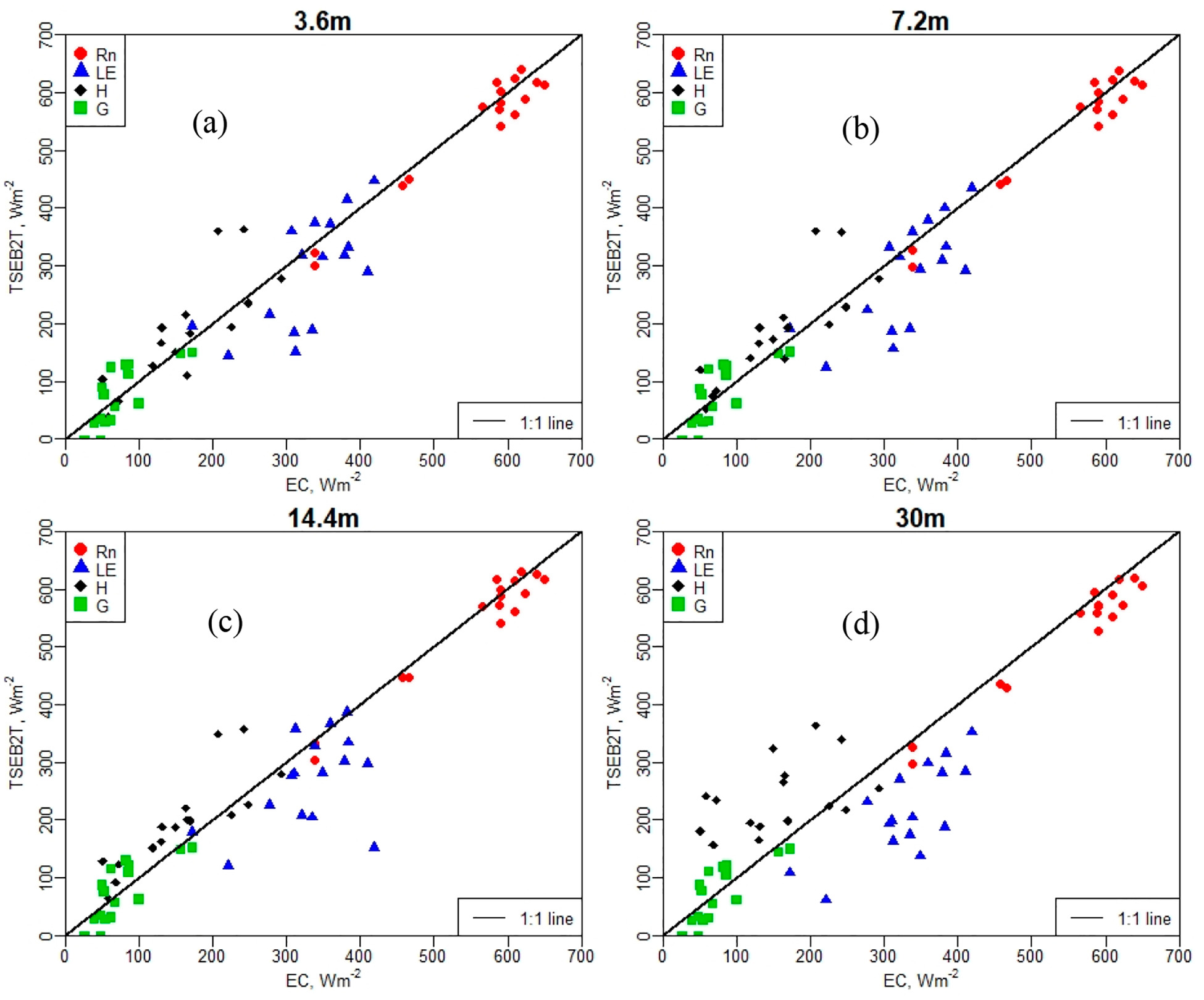
Scatterplot of observed versus estimated surface fluxes using different model grid sizes/resolution with the *TSEB2T* model; (**a**) 3.6 m, (**b**) 7.2 m, (**c**) 14.4 m, and (**d**) 30 m.

Table 3 lists the goodness-of-fit statistics between the energy fluxes using *TSEB2T* at different spatial resolutions and *EC* tower observations, while Figure 6 shows the relationship between the modeled and measured fluxes. The results indicate a significant deterioration in model performance at the 30-m grid size. A major factor that may be responsible for this poor performance in the *TSEB2T* model at 30-m resolution is that the size and dimension of the *EC* source area estimated by the footprint model cannot incorporate a representative range in the spatial variability in the fluxes at 30-m resolution. This conclusion agrees with a previous study conducted by Song et al. [69] that showed a major problem in comparing modeled and measured fluxes when there is a mismatch in pixel resolution or model grid size in the remotely sensed *ET* output and in the source area contributing to the *EC* tower measurements in a heterogeneous landscape.

Results in Table 3 also indicate that *R*_*n*_ and *G* across multiple aggregated grids demonstrated a close agreement between the *TSEB2T* output and observed measurements, as indicated by lower *MAE* and *MAPE* with quite constant correlation (*R*^*2*^). The *MAE* and *MAPE* in the *R*_*n*_ estimate at grid sizes of 3.6 m, 7.2 m, and 14.4 m accounted for less than 25 W/m^2^ and 5%, respectively. However, at Landsat scale the *MAE* increased slightly to 29 W/m^2^. A similar result was obtained for *H*, where *MAE* at the finer resolutions yielded values less than 45 W/m^2^, while the coarser grid size of 30 m yielded a larger *MAE* of nearly 80 W/m^2^. As shown in Table 3, the correlation of *H* is higher than *G* and *LE*, except for 30-m resolution/model grid. This implies that the performance of the 30-m resolution is different compared to the 3.6-m, 7.2-m, and 14.4-m resolutions. The results for *LE* indicated good agreement with the flux measurements at 3.6-m, 7.2-m, and 14.4-m modeling grid sizes, while at the 30-m resolution, the *MAE* value was around 85 W/m^2^. As demonstrated in Figure 6d, all values of *LE* are underestimated (below 1:1 line) with an *NSE* coefficient of 0.2. Furthermore, the highest *NRMSE* values were observed for *LE*, compared with other surface fluxes, particularly at 30-m resolution. The lowest *NRMSE* was obtained for *R*_*n*_ across different spatial domains/model grids.

With the *TSEB2T* model and other remote sensing-based models using thermal-IR as the boundary condition, *LE* is solved as the residual component of the surface energy balance, *LE* = *R*_*n*_ − *H* − *G*. Therefore, an error in the calculation of energy fluxes (*R*_*n*_, *H*, and *G*) adversely affects the estimation of *LE*. Based on Figure 6, the *LE* estimation (or bias) is mainly influenced by the estimation of *H*. This conclusion was also reached by Kustas et al. [70], who showed the discrepancies between modeled and measured *LE* is due in large part, up to approximately 90%, to errors in modeled *H*.

### 3.2. Contextual Spatial Domain Aggregations Effects

#### 3.2.1. The Effect of Model Grid Size on *TSEB2T* Inputs

##### (a) Canopy and Soil Temperatures (*T*_*c*_, *T*_*s*_)

**Figure 7.**
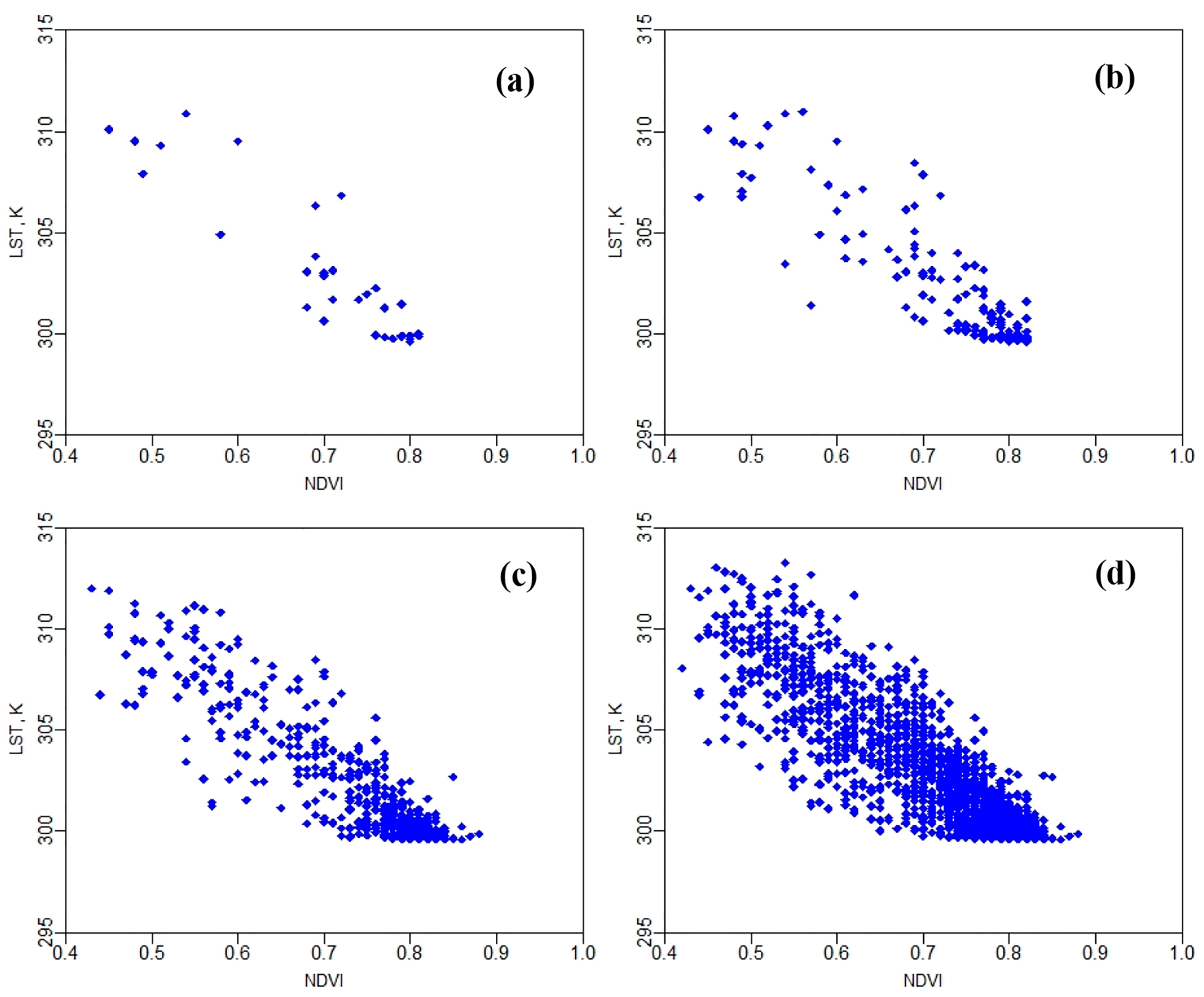
The *LST-NDVI* relationship used for finding *T*_*c*_ and *T*_*s*_ as proposed by the *TSEB2T* model at different spatial domains (09 August 2014). (**a**) 3.6 m, (**b**) 7.2 m, (**c**) 14.4 m, (**d**) 30 m.

*T*_*c*_ and *T*_*s*_ were estimated based on a linear *LST-NDVI* relationship as described by Nieto et al. [2]. However, this relationship does not fulfill the homoscedasticity criterion when the spatial domain/resolution reaches a certain size (i.e., 30-m) as shown in Figure 7. For example, in the case of a 30-m grid size, a higher variability is observed in the *LST-NDVI* data compared with finer resolutions (3.6 m, 7.2 m, and 14.4 m). At micro-scale (e.g., 3.6 m), there are small number of pixels inside the spatial domain compared with others (7.2 m, 14.4 m, and 30 m), and exhibit an apparent linear relationship between *LST* and *NDVI*. However, at coarse resolution (e.g., 30 m), there are many more pixels, more rows of vineyard are included, and large vegetated and bare soil pixels exist inside the spatial domain. The result is a partially filled triangular shape. This indicates the relationship between *LST* and *NDVI* starts to resemble the “triangle method” [71] to estimate *ET* as the sampling domain increases.

**Figure 8.**
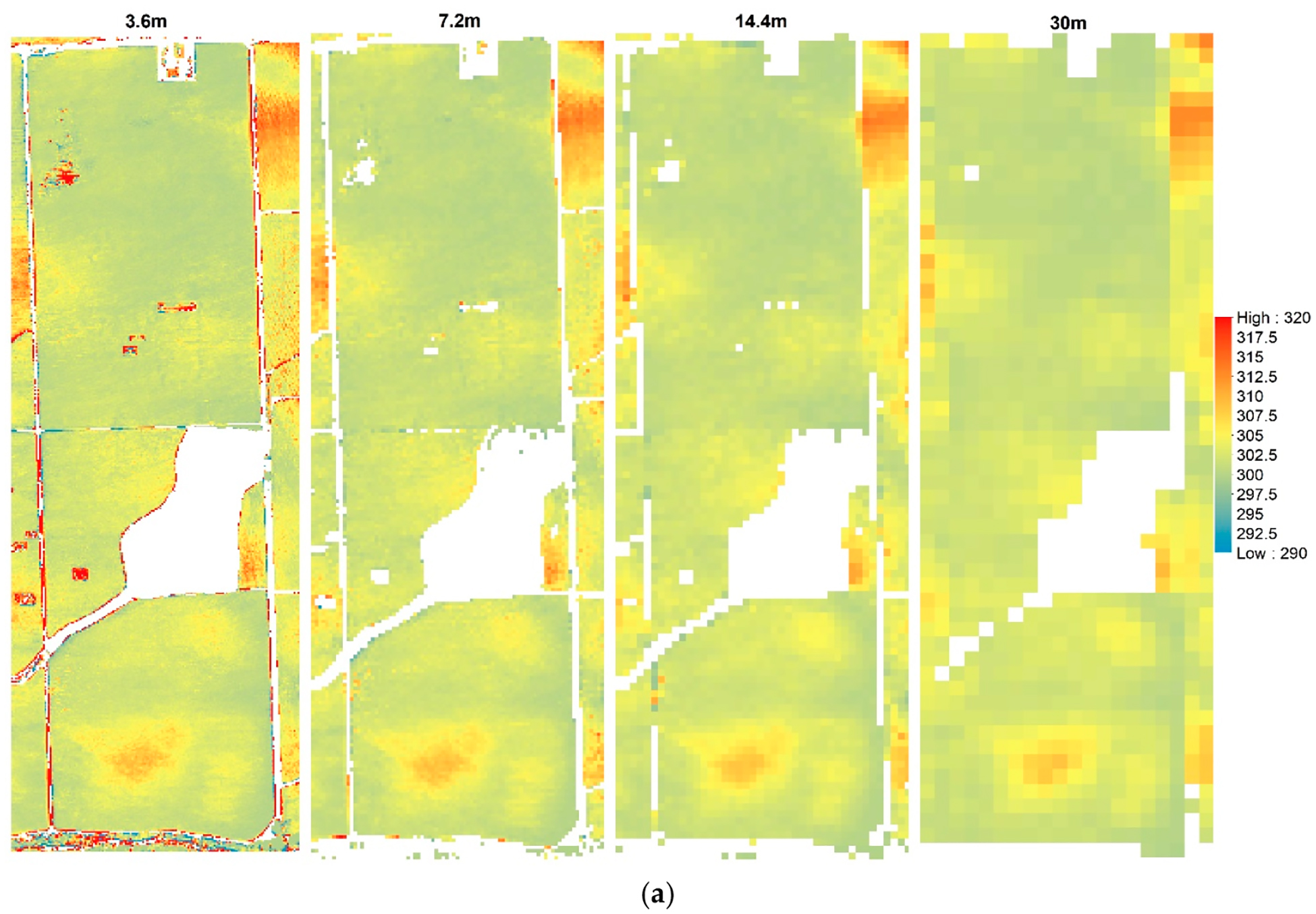

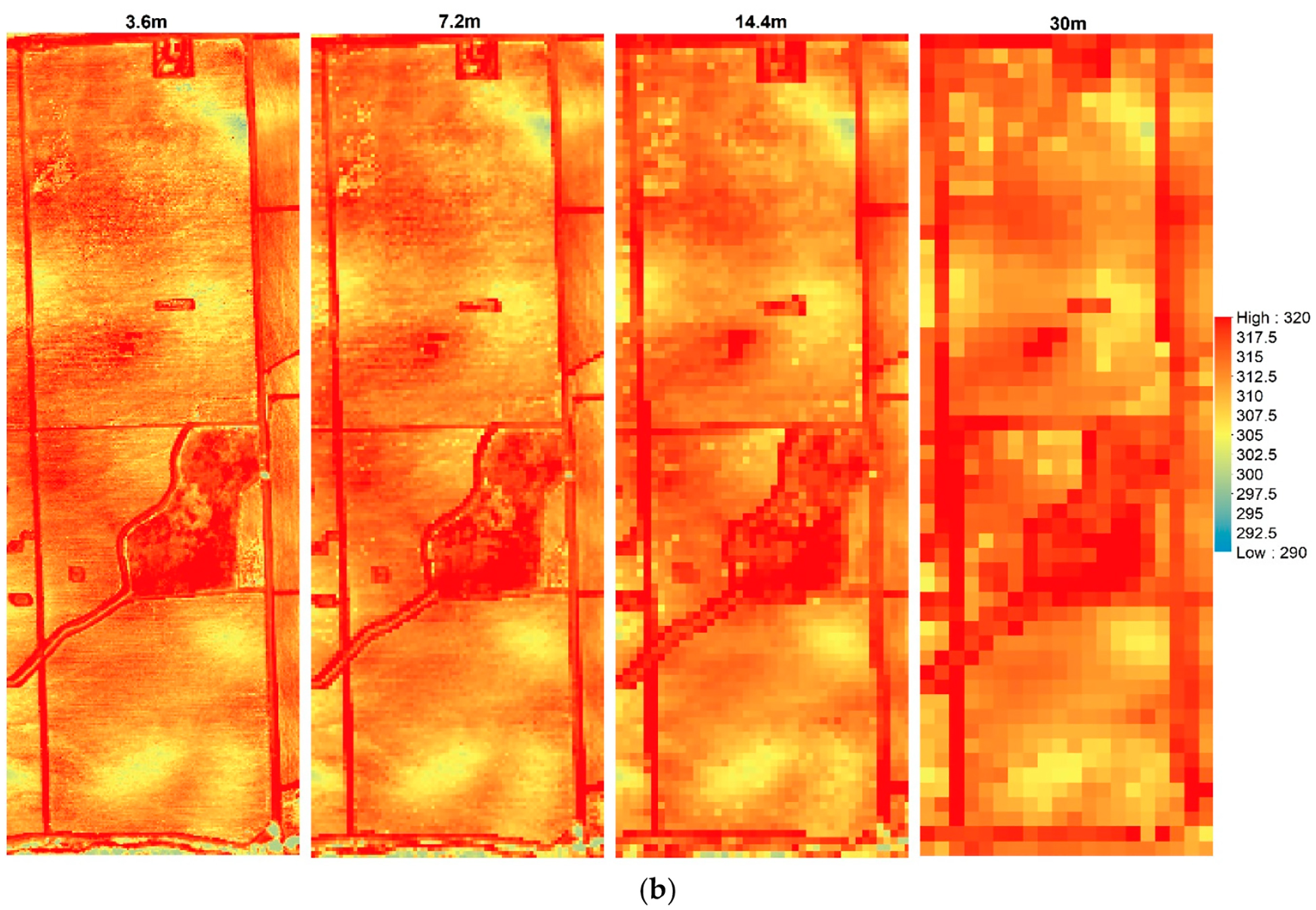
Example of (**a**) canopy temperature (*T*_*c*_) and (**b**) soil temperature (*T*_*s*_) in Kelvin (K) at different spatial domains for 09 August 2014.

Figure 8 illustrates the *T*_*c*_ and *T*_*s*_ maps at different resolutions, which provide an indication of the loss in spatial variability due to spatial aggregation. The ranges of *T*_*c*_ and *T*_*s*_ were between 290 K (16.85 °C) and 320 K (46.85 ° C) for the *sUAS* flight in 2014.

##### (b) Leaf Area Index (*LAI*)

With the *GP* model results, it was found that the main estimators for computing *LAI* are the mean of *NIR*/*R* ratio of the vine, area of the vine, sum of *NDVI* of the vine, standard deviation of *NIR* of the interrow, and standard deviation of *NIR*/*R* ratio of the vine. The *GP* model (Equation (14)) was applied to the remote-sensing imagery to map spatial *LAI* distribution across the study area.

(14) $$LAI=0.21NDVI_{v_{\_}\text{area}}-0.004NDVI_{v_{\_}sum}+0.34{\left(\frac{NIR}{R}\right)}_{v_{\_}\text{mean}}-\frac{0.94}{exp\left(0.23{\left(NDVI_{v_{\_}\text{area}}\right)}^{2}\right)}-2.8NIR_{i{\_}_{STD}}{\left(\frac{NIR}{R}\right)}_{v_{STD}}-0.7$$

*LAI* values from the *GP* model compared with the actual *LAI* field measurements showed good agreement with an *R*^2^ of 0.73.

**Figure 9.**
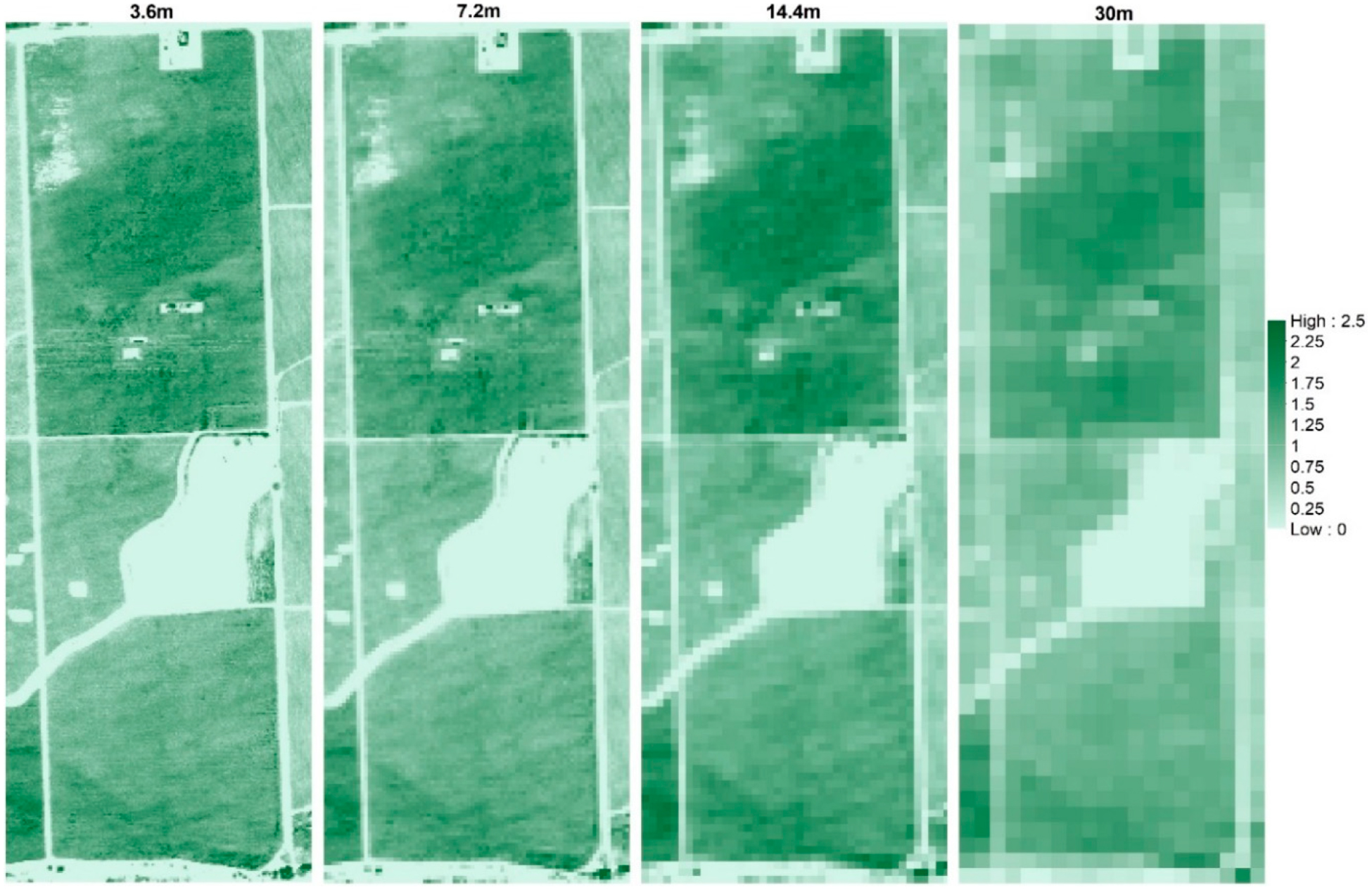
Example of modeled *LAI* (unitless) across different spatial domains for 09 August 2014.

**Figure 10.**
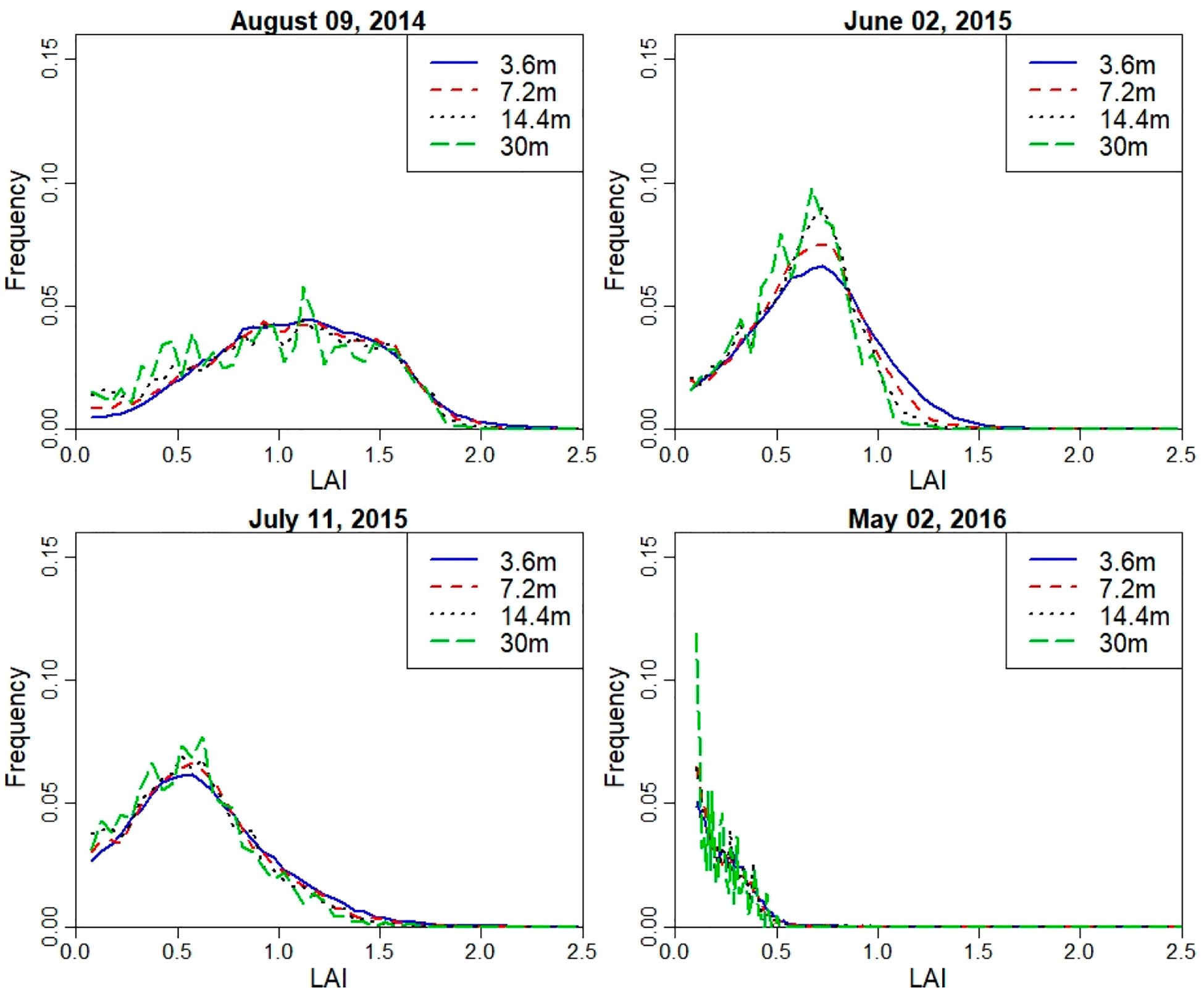
Frequency curve of *LAI* at different times from 3.6 m and 7.2 m, 14.4 m and 30 m.

To evaluate the difference between multiple model grid sizes of *LAI* for each flight, *LAI* maps at different resolutions were estimated (see Figure 9) and statistics including the spatial mean, standard deviation, and coefficient of variation (*CV*) were calculated as shown in Table 4. Figure 9 provides an indication of the loss in spatial variability in *LAI* images due to spatial aggregation. *LAI* at each contextual spatial domain/resolution was calculated using the *LAI* model (Equation (14)). Each parameter in that equation was calculated based on the pixel values inside the model grid. The ranges of *LAI* were between 0 and 2.5 for the *sUAS* flight in 2014. As illustrated in Table 4, the spatial mean value (*μ*) is the same across different scales, with a slight decrease in *CV*. The exception is the flight on 2 May 2016, which represents the early growing stage of the vine canopy with active/live interrow cover crop, showing a higher *CV*. Hardin and Jensen [72] also found greater uncertainty in estimating *LAI* under low *LAI* conditions using *VIs*. The frequency histogram in Figure 10 indicates the distribution of values is skewed such that the lower values are more pronounced for the flight of 2 May 2016, with a non-significant change between curves from the different grid sizes, except the 30-m resolution spatial domain, which shows a higher variation compared with other scales. This behavior aligns with the decreasing *CV* values due to loss in internal or pixel variability of the *LAI* values. A similar trend of lower *CV* toward large scale (30 m) has been observed for other *TSEB2T* inputs including *h*_*c*_, *f*_*c*_, and *w*_*c*_/*h*_*c*_.

#### 3.2.2. Contextual Spatial Domain Effect on Field-Scale *LE* Estimation

**Figure 11.**
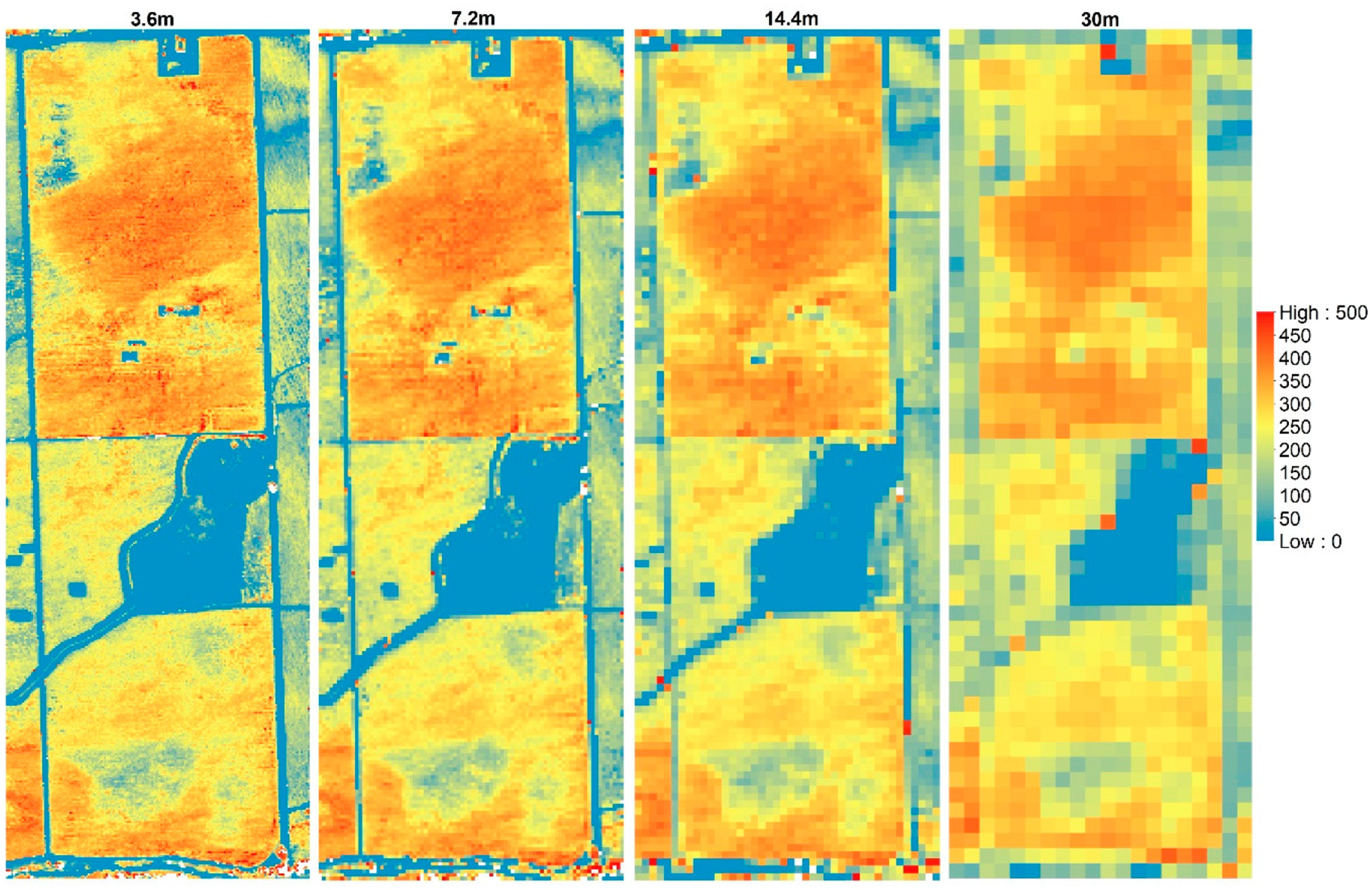
*LE* (W/m^2^) aggregation at 3.6 m, 7.2 m, 14.4 m and 30 m for 09 August 2014.

**Figure 12.**
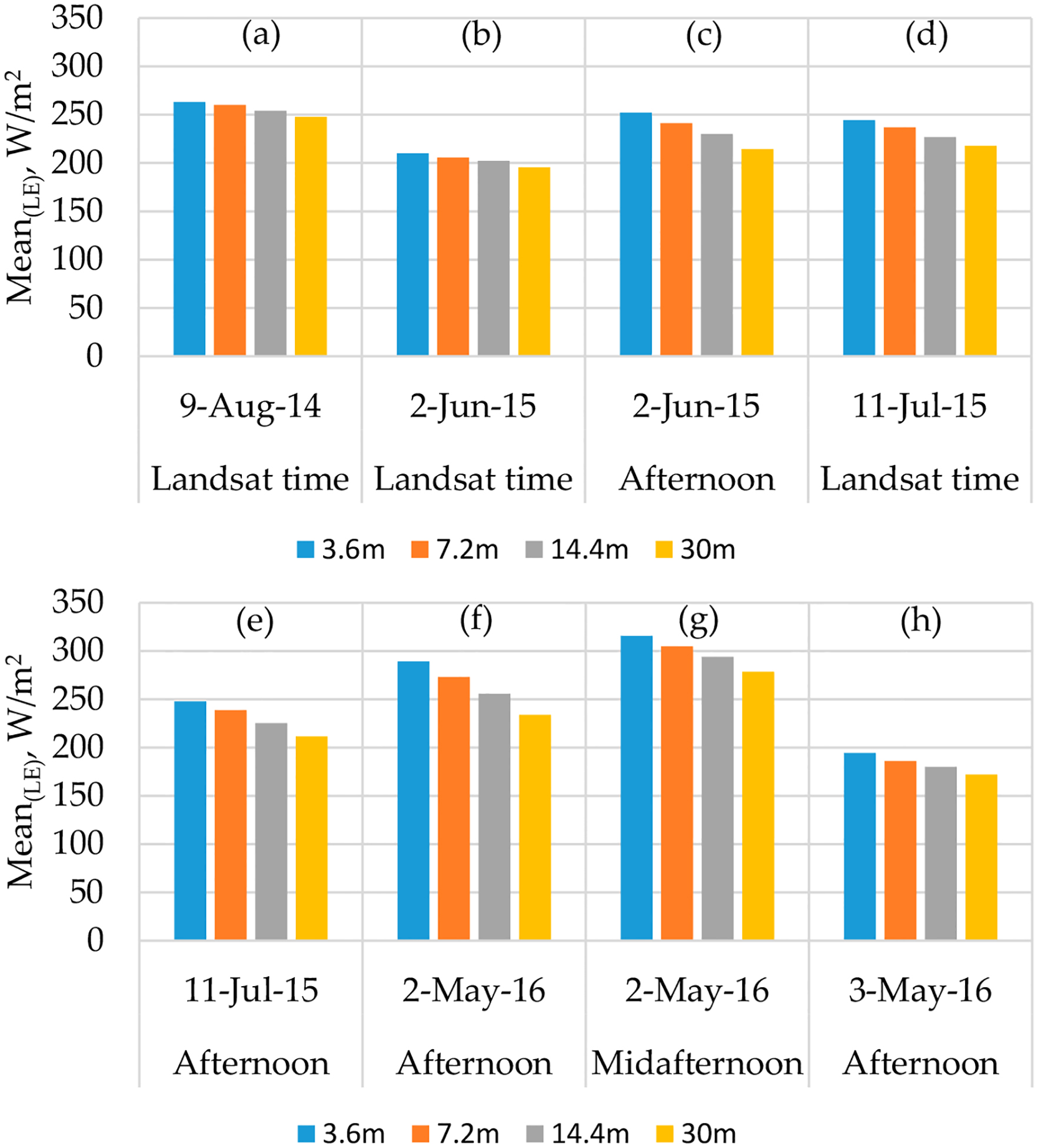
Spatial domain effect on the mean of *LE* spatial data at different times.

**Figure 13.**
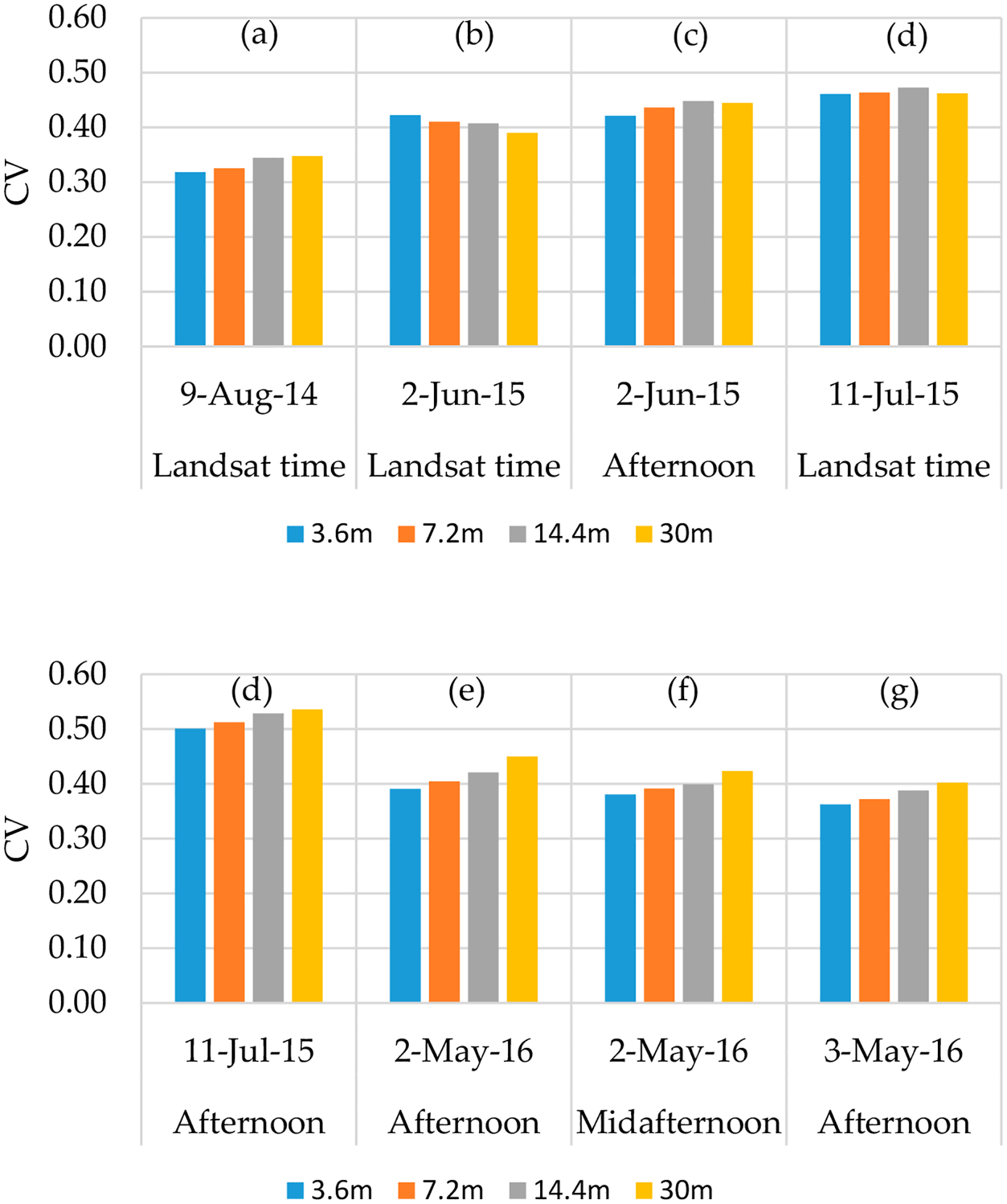
Spatial domain effect on the coefficient of variation (*CV*) of *LE* spatial data at different times.

An example of the maps of *LE* across different model grid sizes is shown in Figure 11. The maps of the energy balance components for 2014 flight at different resolutions are shown in Appendix A. The statistics (mean (*μ*) and coefficient of variation (*CV*)) for the *LE* maps at the different modeling resolutions are illustrated as bar graphs in Figures 12 and 13, respectively. For *LE*, the highest mean value is on 02 May 2016, at midafternoon. Although the grapevine canopy is fully developed by June, *LE* in May at both overpass times is higher than the acquisition in June, July, and August. However, on 3 May, the model yields the lowest *LE* values due to overcast conditions that day significantly reducing incoming solar radiation, and hence, the energy fluxes. The phenocam data (https://hrsl.ba.ars.usda.gov/awhite/CAM/) indicate the high rate of *LE* on 2 May is the result of a rapidly developing vine canopy, together with a transpiring cover crop.

At a contextual spatial domain level, the magnitude of *LE* is degraded as shown in Figure 12 due to the data aggregation from the 3.6-m grid to Landsat scale (30 m). For example, the mean *LE* value from *TSEB2T* on 02 May 2016 at midafternoon was 315 W/m^2^ for the 3.6-m grid decreasing to 304 W/m^2^ for the 7.2-m grid, then decreases further to 293 W/m^2^ and 278 W/m^2^, respectively, for 14.4-m and 30-m grids. As shown in Figure 13, *CV* value slightly increases as the model grid scale/resolution size increases despite a decrease in variation of *LAI* and *LST* distribution as seen in Section 3.2.1. While *LE* degrades, the *CV* values do not show significant differences. This can be due to internal *TSEB2T* compensation of the energy balance components at the different evaluated scales.

#### 3.2.3. Contextual Spatial Domain Effect on *LE* Statistical Characteristics

**Figure 14.**
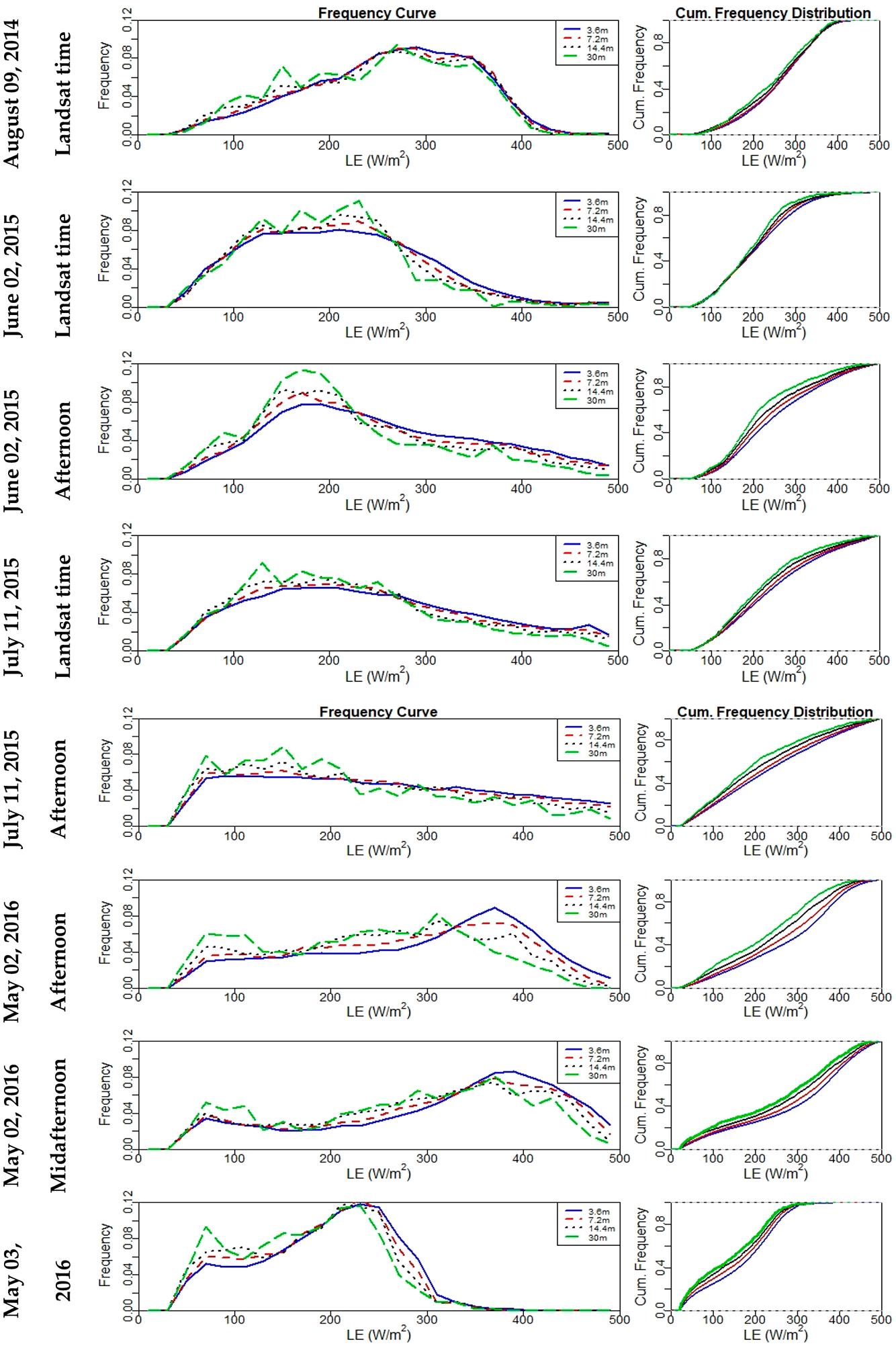
Frequency curve (**left**) and cumulative frequency distribution (**right**) plots of instantaneous *LE* for all *sUAS* flights at 3.6 m, 7.2 m, 14.4 m, and 30 m.

To provide quantitative evaluation of the impact of spatial aggregation of inputs on *LE* estimation for the resulting pixel values, frequency and cumulative distribution plots for the *LE* maps are illustrated in Figure 14. This figure shows that *LE* varies at different grid sizes. The cumulative frequency distribution curves indicate that, especially at the 30-m grid size, *LE* distribution tends to have the highest cumulative values at lower *LE* range (below 300 W/m^2^). A magnitude shift towards lower *LE* persists across different times, with one exception. In the case of a 30-m grid on 02 June 2015, the frequency moved up then decreased below the frequency curves of other grid sizes (3.6 m, 7.2 m, and 14.4 m). In general, the results in Figure 14 show a reduction in *LE* distribution as the scale becomes coarser. Hong et al. [22] indicated that an increase in the peak of the *LE* histogram curve spans as much 10% to 20% as a response to spatial data aggregation using *SEBAL*. In the *TSEB* model, the soil and vegetation components of the scene are treated separately, while the *SEBAL* model uses a single source approach using the composite soil/canopy temperature and is contextual defining wet and dry *ET* limits based on the hot and cold extremes in the *LST* field within the image [73]. Moreover, Ershadi et al. [14] pointed out three possible reasons behind the different results obtained from *ET* models: (a) the approach (e.g., contextual hot/cold surface temperature limits versus using absolute surface-atmosphere temperature differences) of each model to estimate *ET*, (b) the study area and eco-hydrological conditions of the surface, which may favor certain *ET* model parameterizations over others, or (c) the different models of aerodynamic resistance formulations and sensitivity to the roughness parameters.

**Figure 15.**
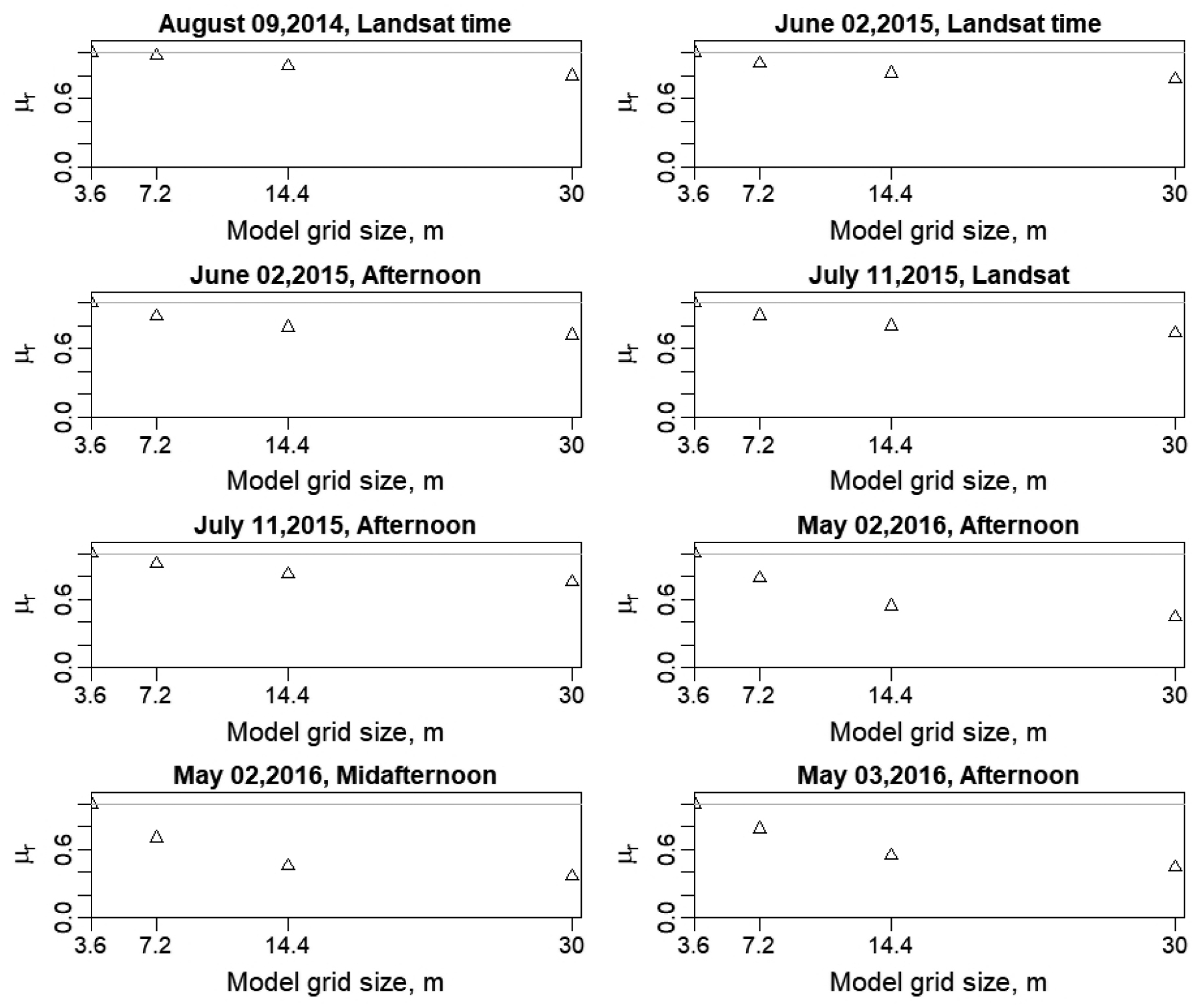
Variation of the relative spatial mean (*μ*_*r*_) of *R*_*a*_ for different flights.

Increasing the spatial domain/resolution affects the estimation of *TSEB2T* parameters as the fine details of the surface disappear. To test these claims, *R*_*a*_ (s/m) and *LST-NDVI* relationship were evaluated at different spatial domain/resolution; the latter is shown in Section 3.2.1 (a). As shown in Figure 15, there is a decreasing trend in the relative spatial mean (*μ*_*r*_) of *R*_*a*_ for all flights, ranging approximately from 20% to 60%. The high variability in *R*_*a*_ is related mainly to the variables that affect the friction velocity (u_*_), which the mean canopy height and roughness length (*z*_*oH*_), which are derived from the imagery at different resolution/spatial domain. This finding is in agreement with Ershadi et al. [14] and Moran et al. [15], who indicated that the reduction of *R*_*a*_ value at coarse spatial domain/resolution is a key factor behind the underestimation of *LE*.

#### 3.2.4. Effects of Model Grid Size on *LE*

**Figure 16.**
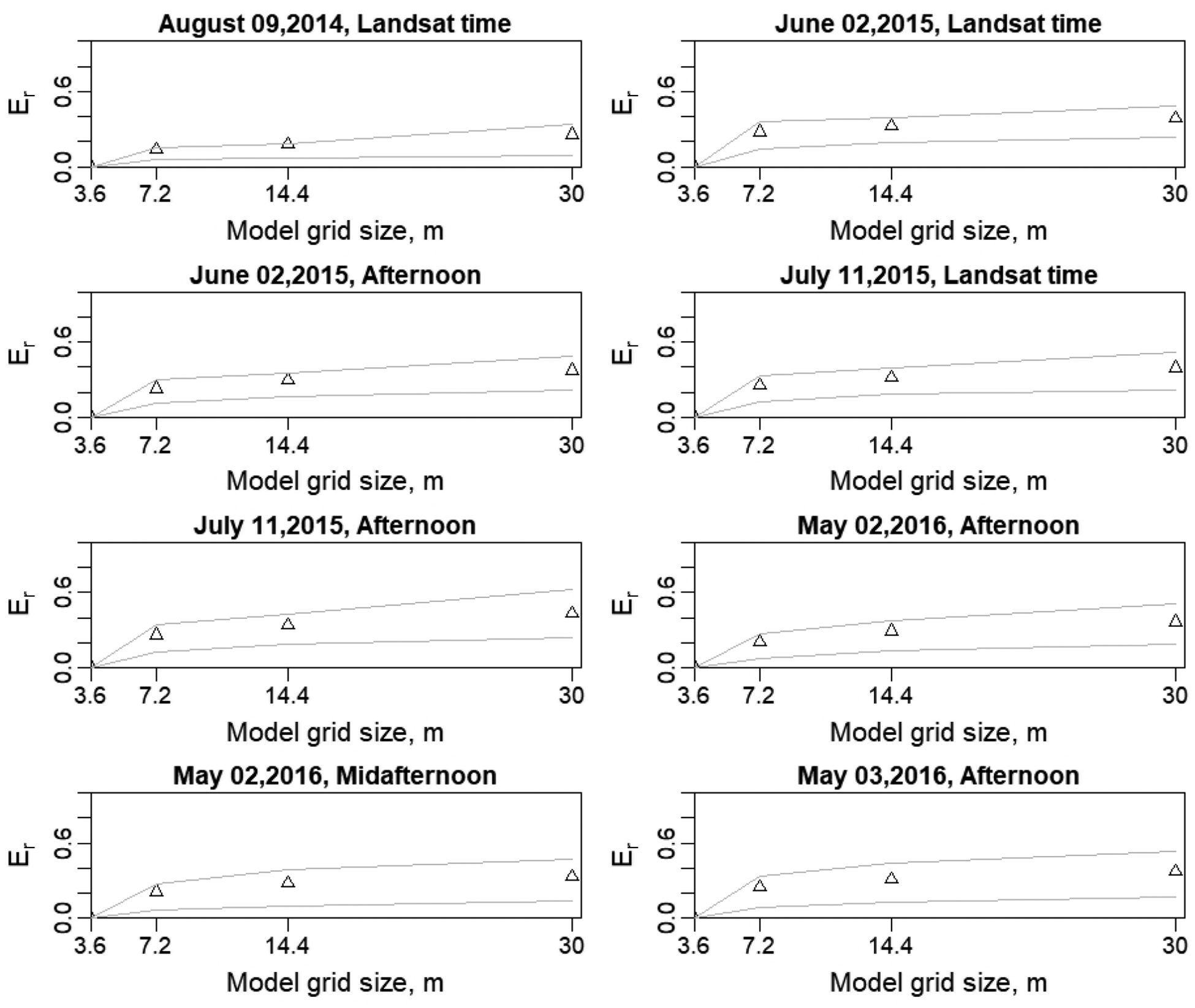
Relative error (*E*_*r*_) at different spatial resolutions for *LE* with the triangle symbols indicating mean and light lines indicating the 25th and 75th percentiles for the coarse grid sizes.

To evaluate quantitatively the impact of model grid size via the resolution of key input data, the relative difference (relative error) (*E*_*r*_) was computed using as the reference the *LE* at 3.6-m model grid size/resolution. For example, the *LE* value at the 7.2-m grid is compared to the *LE* at the 3.6-m grid size by resampling the 7.2-m grid to a 4 × 4 set of 3.6-m *LE* output which will have a uniform *LE*-value at the finer resolution, and taking the difference. As illustrated in Figure 16, *E*_*r*_ is calculated with the mean and percentiles (25th and 75th) for the coarser grid sizes used in the *TSEB2T* model for the different *sUAS* acquisitions. The plots demonstrate an increasing trend in *E*_*r*_ as the model grid size/resolution increases/decreases. The largest *E*_*r*_ value was computed for the imagery on 11 July 2015 at afternoon at nearly 45% for the Landsat resolution. In contrast to 11 July 2015, the lowest range of relative error was observed on 09 August 2014, where the *E*_*r*_ ranged approximately between 15% for the 7.2-m grid and 25% for the 30-m grid. On an average, *E*_*r*_ value ranged from approximately 25% using the 7.2-m model grid size to 40% with the 30-m model resolution.

These results are supported by an Ershadi et al. [14] study that found the *E*_*r*_ of *LE* varied between 20% and 40% when aggregating the Landsat data incrementally from 120 m to 960 m and using the *SEBS* model to calculate surface heat fluxes. Furthermore, Moran et al. [15] indicated that a larger error could appear (larger than 50%) in *H* estimation over a heterogeneous area due to a mix of stable and unstable conditions and the variation in aerodynamic roughness, especially for highly unstable conditions. As previously mentioned in Section 3.1, the underestimated *LE* could be influenced by overestimation in *H*, which implies that a large error is expected in the residual flux (*LE*) estimate at coarse spatial domains [70]. Furthermore, the effect of model grid size on *LE* is also visible at the 25th and 75th percentiles, which immediately increases at the 7.2-m grid size and continues increasing towards the 30-m resolution, providing a clear indication of increasing discrepancy with the reference grid (3.6 m) *LE* estimates.

## 4. Conclusions

The objective of this study was to assess high-resolution *LE* estimation in vineyards at different model grid sizes or resolutions, specifically 3.6 m, 7.2 m, 14.4 m, and 30 m (Landsat scale), using a physically-based *ET* model known as *TSEB2T*. The reference grid size of 3.6 m represents the finest pixel resolution that includes both vine canopy and interrow conditions, which is the resolution where the *TSEB* model algorithms of soil/substrate and canopy temperature partitioning radiation and convective energy exchange are applicable [2]. Multiple statistical measures were used to assess the effect of decreasing the spatial resolution or increasing the model grid size 2, 4, and nearly 10 times the original 3.6 m resolution. These included validation of *TSEB2T* fluxes at the different model grid sizes with the *EC* measurements, comparing *LE* spatial statistics (mean and coefficient of variation, frequency distributions) and *LE* differences over the imaged domain at the different resolutions using *LE* at 3.6 m grid size as the reference. The results showed that separation of *T*_*c*_ and *T*_*s*_, required in *TSEB2T*, affects the *LST-NDVI* linear trend as a function of resolution of the pixels. The validation results with the flux tower measurements indicate that *R*_*n*_ and *G* discrepancies do not change across different model grid sizes, while for *H* and *LE* there is an increase in model-measurement differences, particularly at the 30-m resolution. This is largely caused by an overestimation in *H*, causing an underestimation in *LE* (bias), particularly at the coarsest resolution (30-m grid size). This refers mainly to the non-linear relationship of *LST-NDVI* and the variability of *R*_*a*_ due to the variables that affect the u_*_ which are the mean canopy height and roughness length, which are derived from remote sensing imagery at different spatial domain/resolution.

The effects of model grid size were evaluated at field and at grid scale using the spatial mean and coefficient of variation and relative difference, respectively. At field scale, the results show small decreases in the spatial mean over the image, ranging approximately from 10% to 20%, as the data aggregated for model grid size increased from 3.6 m to 30 m. However, the relative differences with resolution indicate a significant decrease in *LE*, ranging approximately from 25% to 45%, when aggregating the data from 3.6 m to Landsat scale (30 m). This means that, while field values of *LE* may be adequate to use, the field variability reduction limits its use for precision agriculture applications, such as identifying areas within the field under actual stress conditions or being over irrigated. These results suggest that *TSEB2T* is only applicable using imagery with high enough resolution that can readily distinguish plant canopy and soil/substrate temperatures and the modeling grid size is at a resolution where it is appropriate to apply *TSEB2T* algorithms for modeling the radiative and convective energy exchange from both the vegetation and soil substrate systems. Aggregating inputs to *TSEB2T* to multiple grid sizes of the interrow/row spacings for vineyards is not advisable, since it is likely the accuracy of surface fluxes, particularly *LE*, will deteriorate. While this study was limited to evaluating different modeling grid sizes, a future comparison with Landsat and ECOstress *ET* products is also planned, which would provide a more comprehensive scaling assessment of *ET* estimates for *sUAS*-Satellite *ET* integration. Furthermore, the effect of remote sensing resolution on the output of other *TSEB* versions such as *TSEB-PT* may be less affected and will be evaluated in a future study.

## Figures and Tables

**Table 1.** Dates and times of AggieAir *GRAPEX* flights used in this study.

Flight Date	Landsat Time PST	Afternoon PST	Midafternoon PST
09 August 2014	10:41 am		-
02 June 2015	10:43 am	14:07 pm	-
11 July 2015	10:35 am	14:14 pm	
02 May 2016	-	12:05 pm	15:04 pm
03 May 2016	-	12:48 pm	-

**Table 2.** Description of in-situ micrometeorological measurements in this study.

ID	Micrometeorological Parameters	Instrument Name^1^	Elevation
1	Water vapor concentration	Infrared gas analyzer (EC150, Campbell Scientific, Logan, Utah)	5 m agl
2	Wind velocity	Sonic anemometer (CSAT3, Campbell Scientific)	5 m agl
3	Net radiation	4-way radiometer (CNR-1, Kipp and Zonen, Delft, The Netherlands)	6 m agl
4	Air temperature	Gill shielded temperature (Vaisala, Helsinki, Finland)	5 m agl
5	Water vapor pressure	Humidity probe (HMP45C, Vaisala, Helsinki, Finland)	5 m agl
6	Soil heat flux	Five plates (HFT-3, Radiation Energy Balance Systems, Bellevue, Washington)	−8 cm
7	Soil temperature	Thermocouples	−2 cm
8	Soil moisture	Soil moisture probe (HydraProbe, Stevens Water Monitoring Systems, Portland, Oregon)	−5 cm

1 The use of trade, firm, or corporation names in this article is for the information and convenience of the reader. Such use does not constitute official endorsement or approval by the US Department of Agriculture or the Agricultural Research Service of any product or service to the exclusion of others that may be suitable.

**Table 3.** Goodness-of-fit statistics between the eddy covariance and the *TSEB2T* fluxes at different spatial scales (3.6 m, 7.2 m, 14 m, and 30 m).

Spatial Domain	Fluxes	RMSE (W/m^2^)	NRMSE	MAE (W/m^2^)	MAPE (%)	NSE	R^2^
**3.6 m**	*R*_*n*_	28	0.3	25	5	0.9	0.94
	*LE*	69	1.2	58	20	0.5	0.49
	*H*	54	0.8	41	26	0.7	0.67
	*G*	34	0.9	30	51	0.6	0.56
**7.2 m**	*R*_*n*_	27	0.3	24	4	0.9	0.94
	*LE*	66	1.2	56	19	0.5	0.53
	*H*	51	0.7	36	24	0.7	0.67
	*G*	33	0.8	30	50	0.6	0.58
**14.4 m**	*R*_*n*_	25	0.3	20	4	0.9	0.95
	*LE*	79	1.4	56	18	0.1	0.21
	*H*	48	0.7	35	26	0.6	0.69
	*G*	32	0.8	29	49	0.6	0.59
**30 m**	*R*_*n*_	34	0.4	29	5	0.9	0.96
	*LE*	101	1.8	86	30	0.2	0.53
	*H*	93	1.3	78	67	−0.1	0.23
	*G*	31	0.8	28	48	0.6	0.60

**Table 4.** Spatial domain effect on *LAI* estimation.

Flight	Spatial Domain	*μ*	*σ*	*CV*
**09 August 2014**	3.6 m	0.91	0.56	0.61
	7.2 m	0.91	0.54	0.59
	14.4 m	0.91	0.52	0.57
	30.0 m	0.91	0.48	0.53
**02 June 2015**	3.6 m	0.97	0.38	0.66
	7.2 m	0.97	0.33	0.58
	14.4 m	0.97	0.30	0.52
	30.0 m	0.97	0.27	0.47
**11 July 2015**	3.6 m	0.52	0.39	0.75
	7.2 m	0.92	0.36	0.69
	14.4 m	0.92	0.34	0.65
	30.0 m	0.92	0.31	0.60
**02 May 2016**	3.6 m	0.06	0.11	1.90
	7.2 m	0.06	0.10	1.75
	14.4 m	0.06	0.10	1.66
	30.0 m	0.06	0.09	1.59

**Figure A1.**
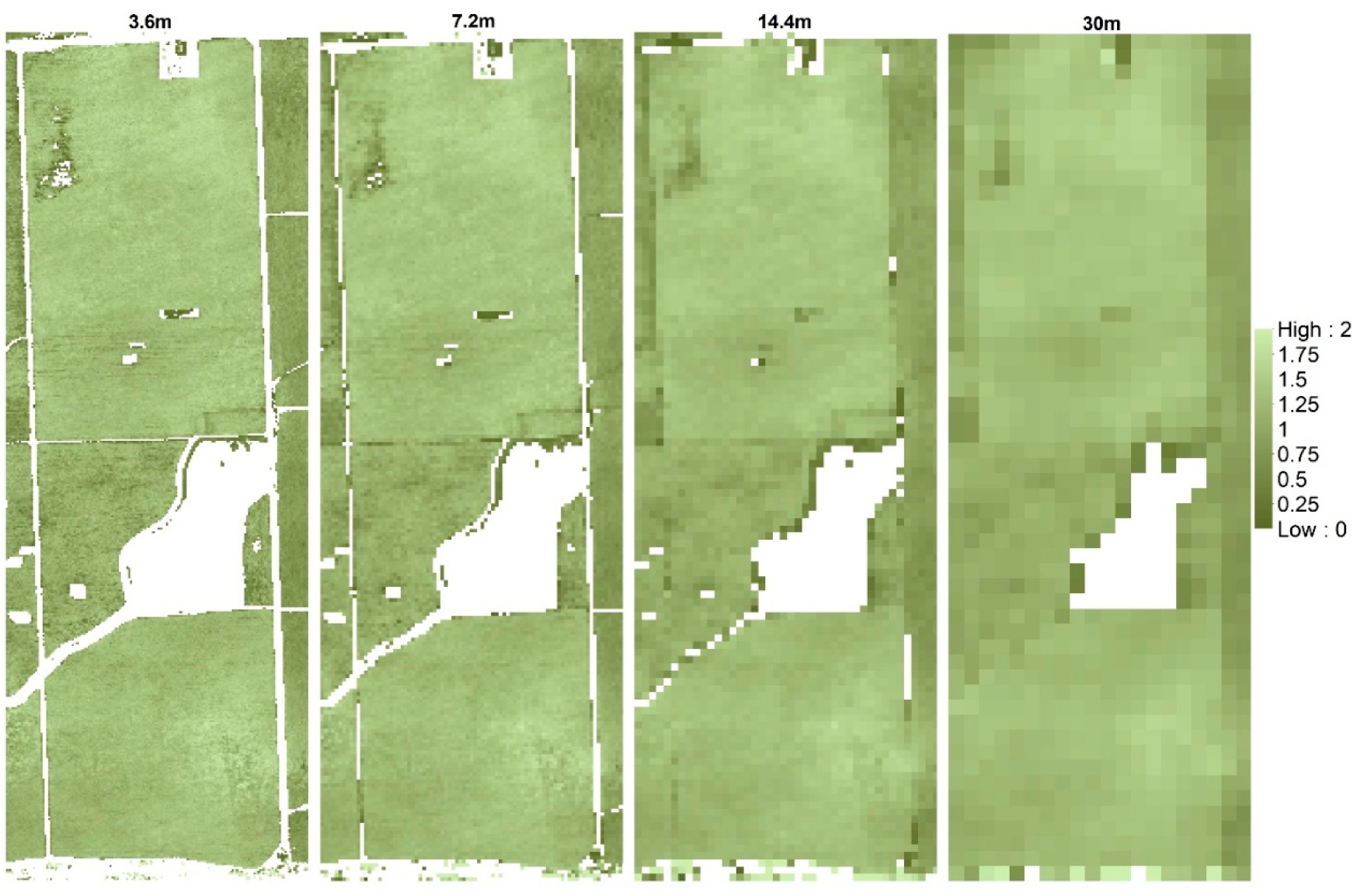
Example of modeled *h*_*c*_ (m) across different spatial domains for 09 August 2014.

**Figure A2.**
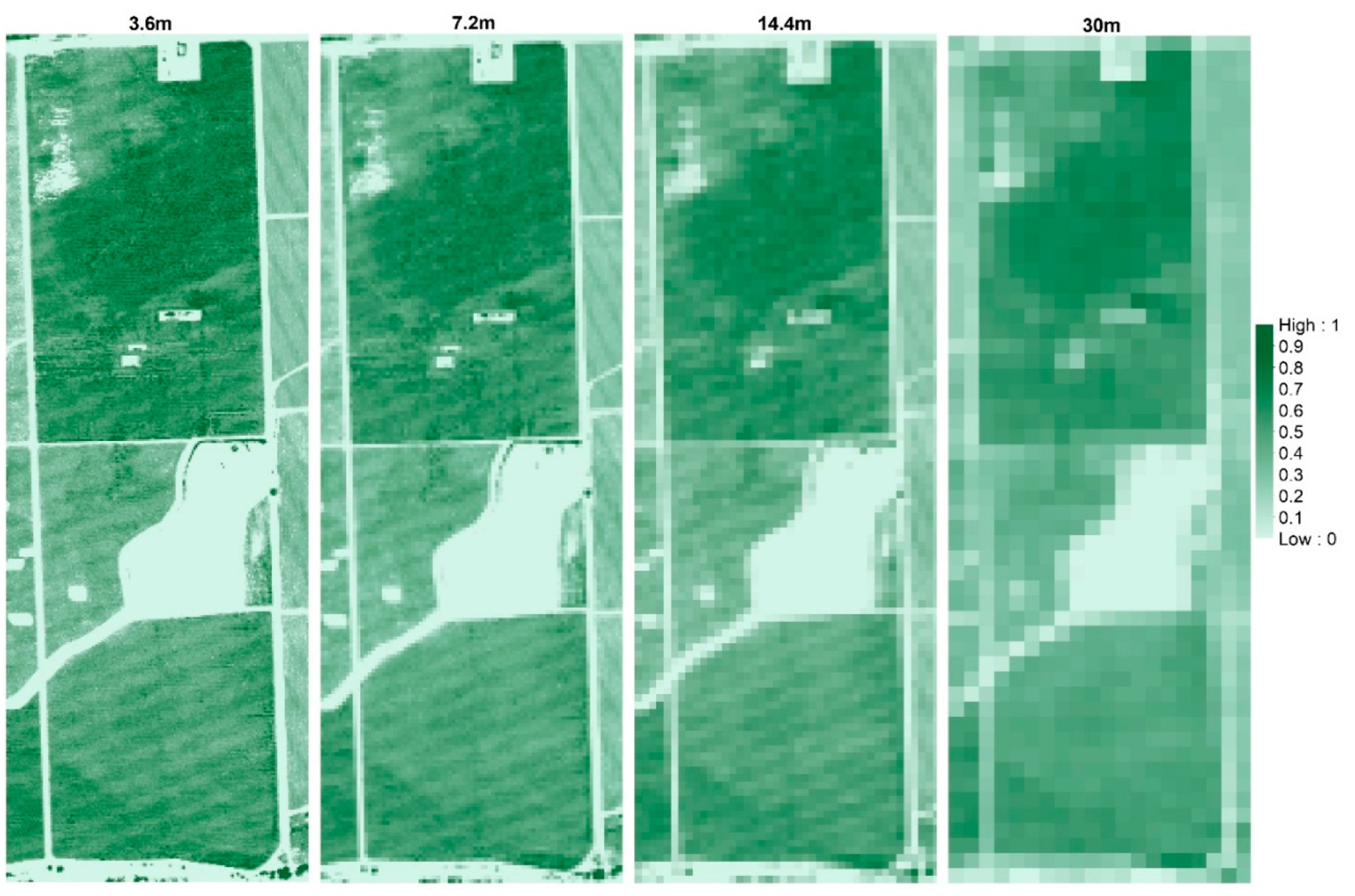
Example of modeled *f*_*c*_ across different spatial domains for 09 August 2014.

**Figure A3.**
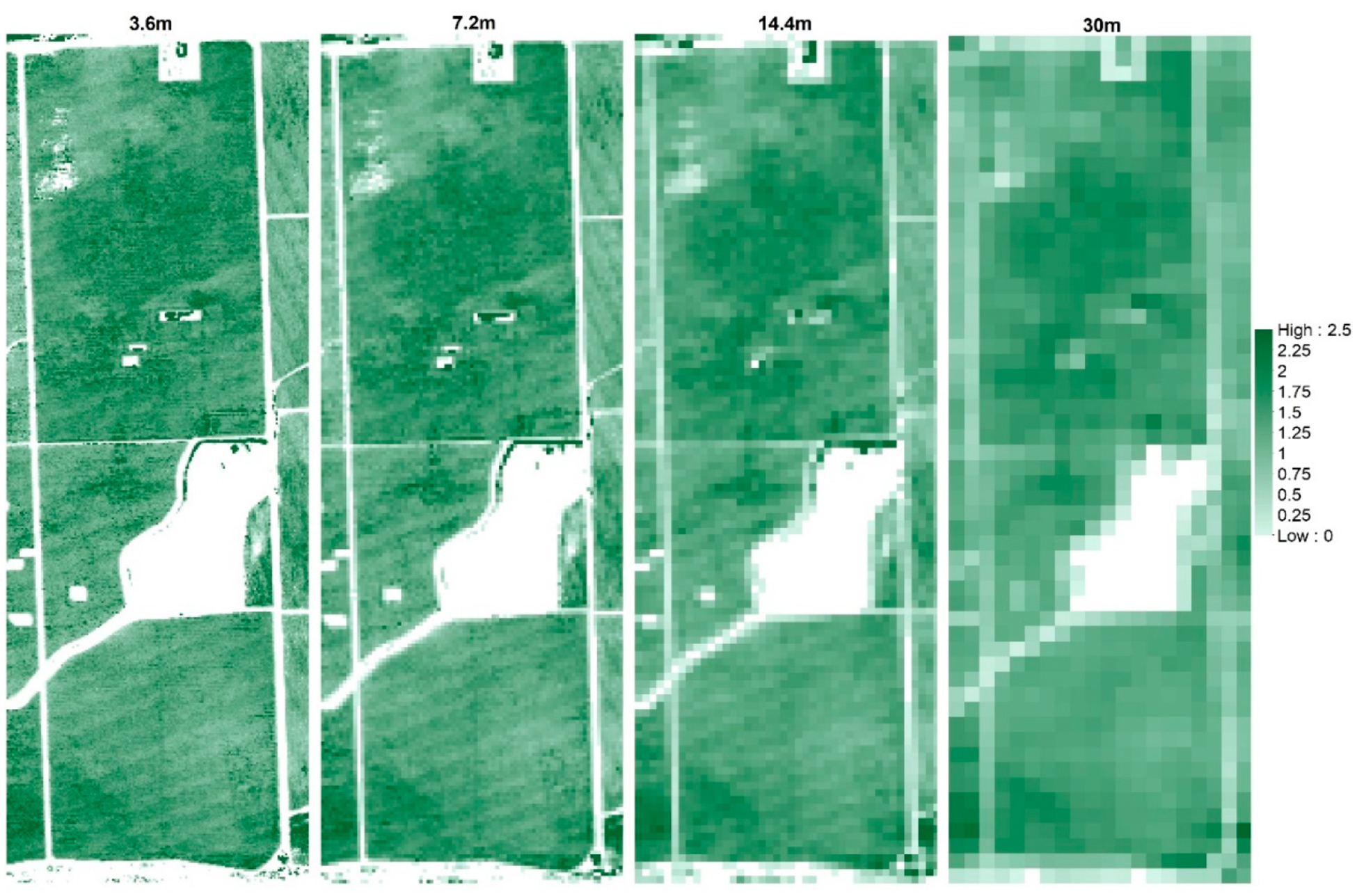
Example of Modeled *w*_*c*_/*h*_*c*_ different spatial domains for 09 August 2014.

**Figure A4.**
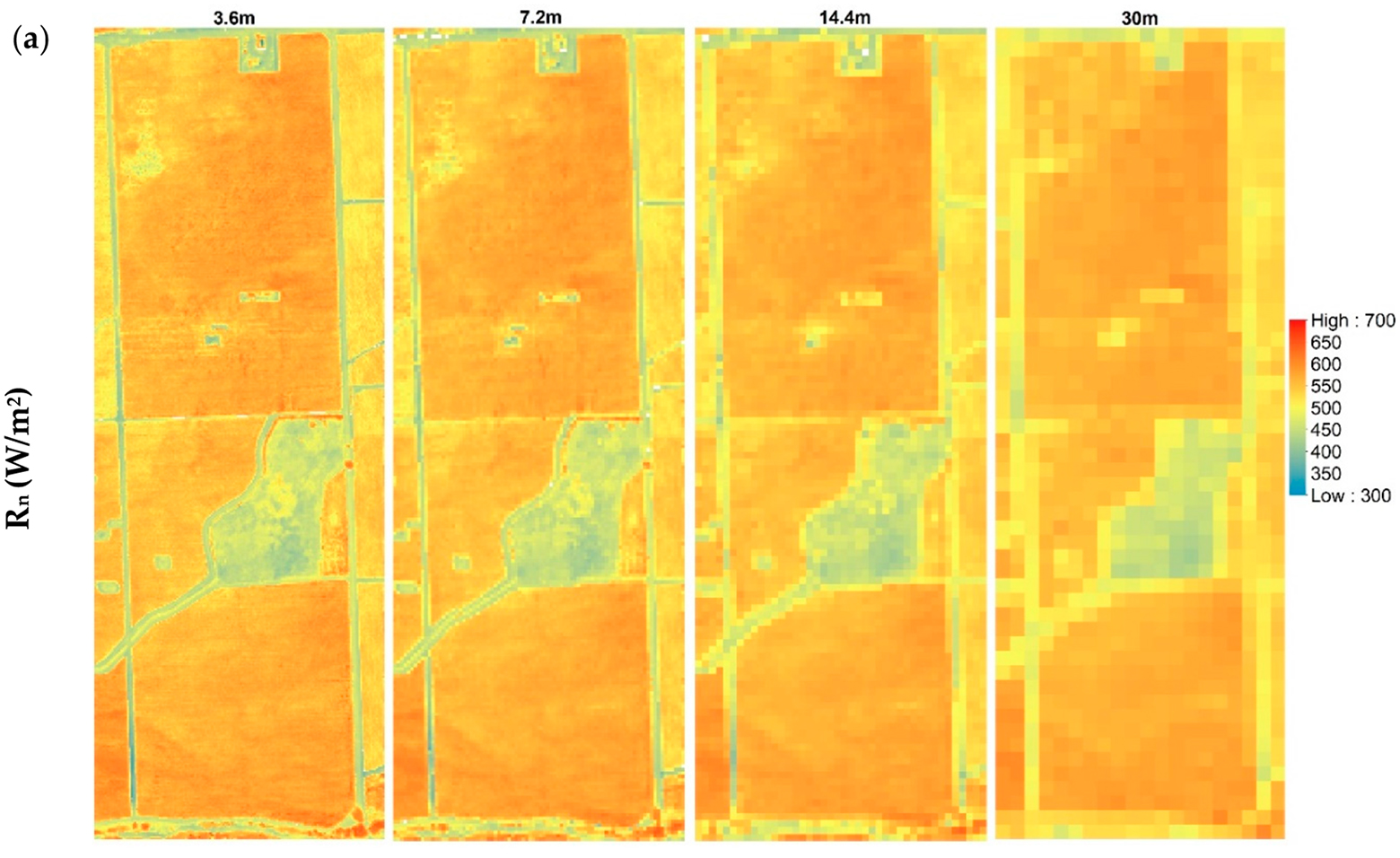

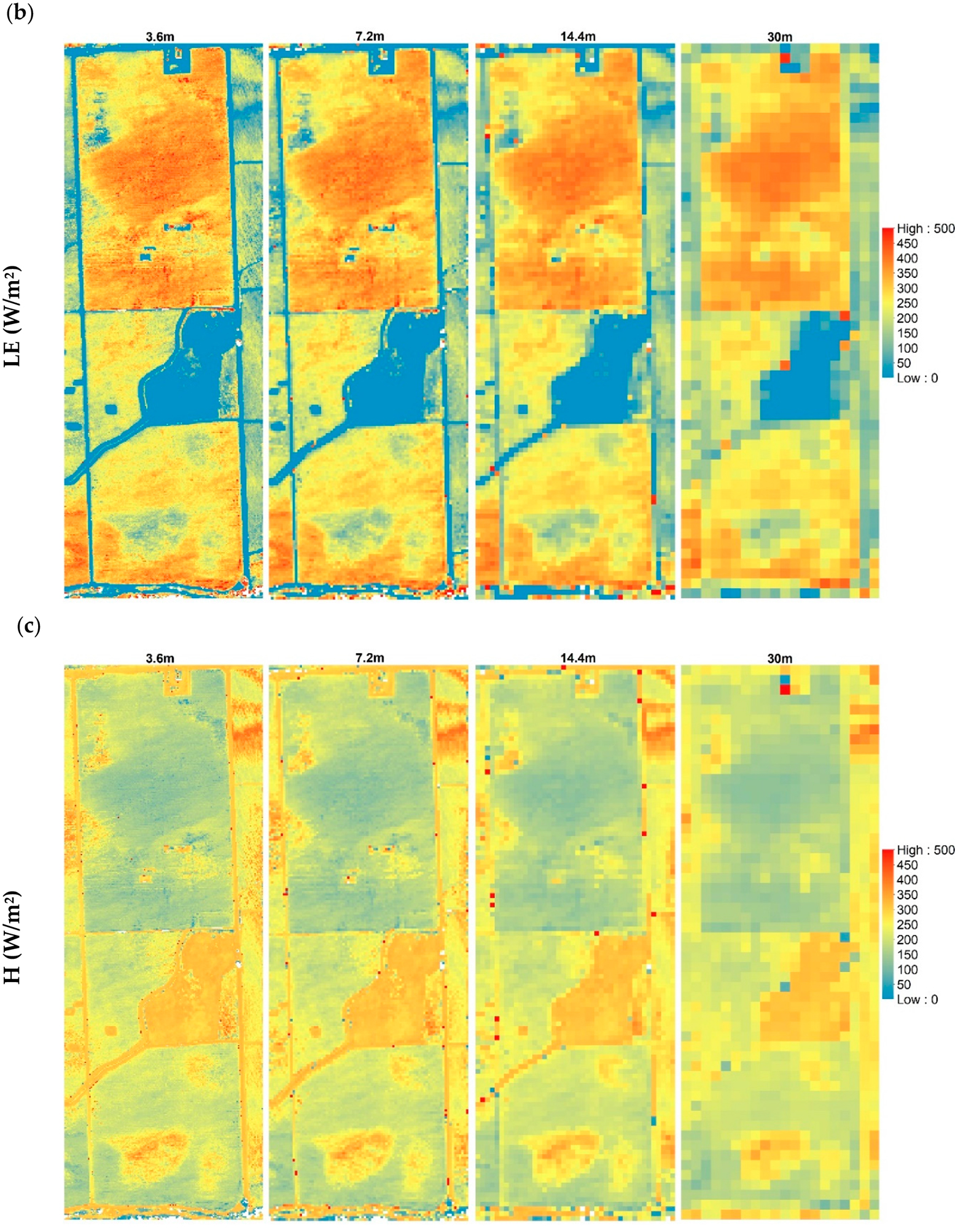

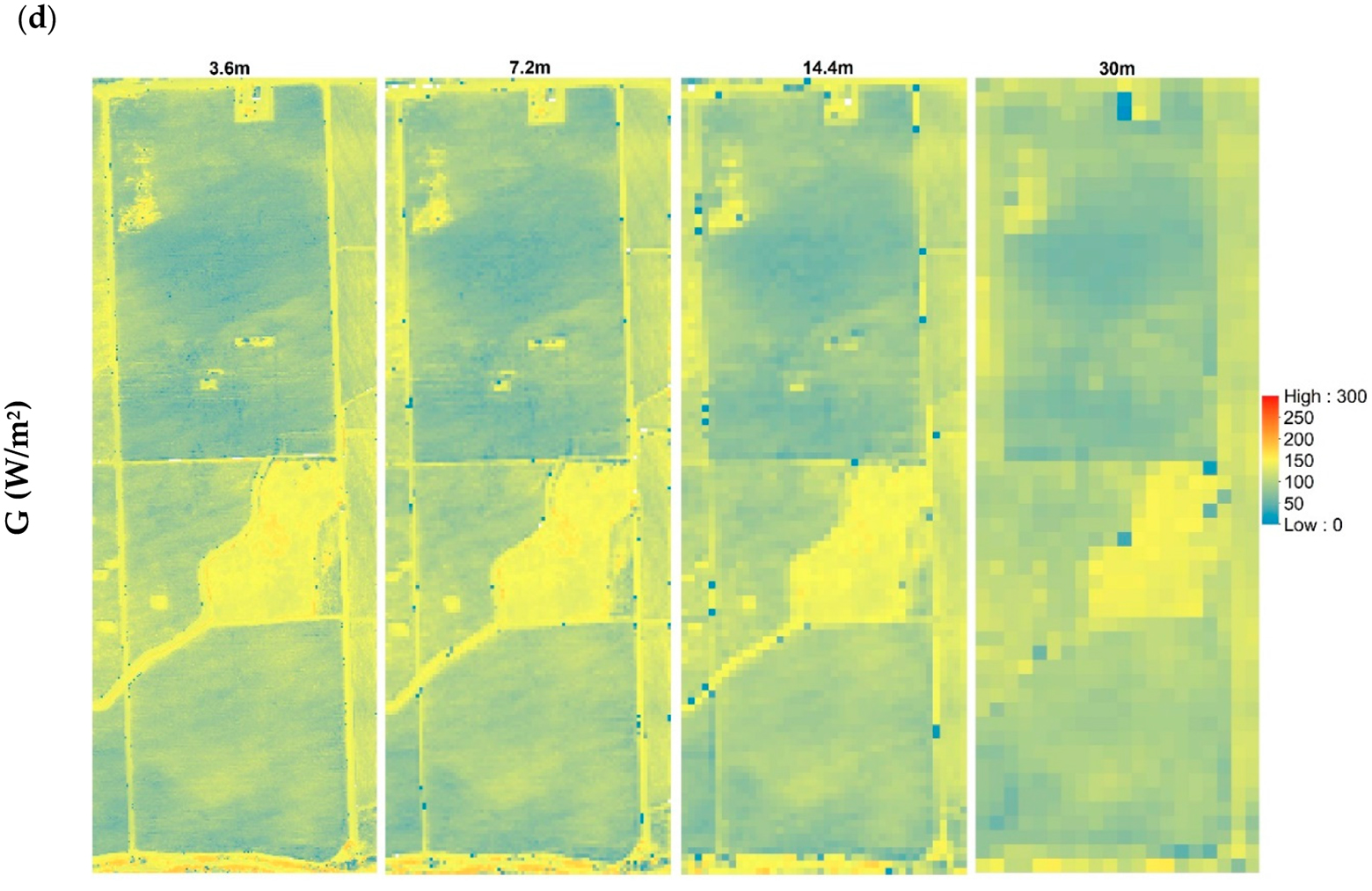
Aggregation of surface energy fluxes across different spatial domain (3.6 m, 7.2 m, 14.4 m, and 30 m) on 09 August 2014: (**a**) *R*_*n*_, (**b**) *LE*, (**c**) *H*, and (**d**) *G*.
